# Insights into the historical assembly of global dryland floras: the diversification of Zygophyllaceae

**DOI:** 10.1186/s12862-018-1277-z

**Published:** 2018-11-09

**Authors:** Sheng-Dan Wu, Lin-Jing Zhang, Li Lin, Sheng-Xiang Yu, Zhi-Duan Chen, Wei Wang

**Affiliations:** 1 State Key Laboratory of Systematic and Evolutionary Botany, Institute of Botany, Chinese Academy of Sciences, 20 Nanxincun, Xiangshan, Beijing, 100093 China; 2 College of Life Sciences, Shanxi Normal University, 1 Gongyuan, Yaodu, Linfen, 041000 China; 3 University of Chinese Academy of Sciences, 19 Yuquan Road, Beijing, 100049 China

**Keywords:** Drylands, Climate change, Divergence time, Diversification rate, Miocene, Phylogeny

## Abstract

**Background:**

Drylands cover nearly 41% of Earth’s land surface and face a high risk of degradation worldwide. However, the actual timeframe during which dryland floras rose on a global scale remains unknown. Zygophyllaceae, an important characteristic component of dryland floras worldwide, offers an ideal model group to investigate the diversification of dryland floras. Here, we used an integration of the phylogenetic, molecular dating, biogeographic, and diversification methods to investigate the timing and patterns of lineage accumulation for Zygophyllaceae overall and regionally. We then incorporated the data from other dominant components of dryland floras in different continents to investigate the historical construction of dryland floras on a global scale.

**Results:**

We provide the most comprehensive phylogenetic tree for Zygophyllaceae so far based on four plastid and nuclear markers. Detailed analyses indicate that Zygophyllaceae colonized Africa, Asia, Australia, and the New World at different periods, sometimes multiple times, but Zygophyllaceae lineages in the four regions all experienced a rapid accumulation beginning at the mid-late Miocene (~ 15–10 Ma). Other eleven essential elements of dryland floras become differentiated at the same time.

**Conclusions:**

Our results suggest that the rise of global dryland floras is near-synchronous and began at the mid-late Miocene, possibly resulting from the mid-Miocene global cooling and regional orogenetic and climate changes. The mid-late Miocene is an essential period for the assembly and evolution of global dryland floras.

**Electronic supplementary material:**

The online version of this article (10.1186/s12862-018-1277-z) contains supplementary material, which is available to authorized users.

## Background

Drylands form nearly 41% of Earth’s land surface [1] and are by definition areas where precipitation is scarce (≤ 250 mm/y) and typically of high variability [2, 3]. They are mainly distributed in tropical and subtropical dryland zones (0–30° latitude), occurring in Africa, Australia, and the New World, and in temperate dryland zones (> 30° latitude), largely limited to the Asian interior and western North America [4]. These dryland regions are home to more than 38% of the world’s population [5] and harbor rich biodiversity [4, 6]. Due to climate change and human activity, drylands worldwide face a high risk of degradation [3, 7]. The remarkable characteristic of dryland floras is a tight coupling of water availability and vegetation dynamics [8]. Compared with our knowledge regarding tropical/temperate forest and arctic regions [9–11], information about the main evolutionary processes underlying species richness in dryland floras is limited.

Geologic sediments indicate that the first onset of arid conditions in Africa and the New World occurred during the early Tertiary period [12, 13]. The aridification in the Asian interior first occurred near the Eocene-Oligocene boundary ~ 34 million years ago (Ma) [14]. Aridification in Australia began in the mid-Miocene, ~ 15 Ma [15]. Thus, the initial aridity in different continents was non-synchronous, which could suggest that the establishment of modern dryland floras in different continents was non-contemporaneous. The global cooling process between the Miocene and Pliocene also enhanced aridity on a global scale [13, 16]. The relation between the evolution of dryland floras and the cooling was not well understood.

In recent years, increasing numbers of studies have focused on the evolutionary history of arid-adapted plants, contributing greatly to our understanding of the assembly and evolution of dryland floras. Southern African ice plants (Aizoaceae), an important and characteristic component in the succulent biome of Africa, are dated to ~ 26 Ma and have diversified rapidly between 8.7 to 3.8 Ma [17]. *Agave* (Agavaceae), keystone plants of semiarid to arid regions in North American deserts, originated ~ 10 Ma and experienced two pulses of increased diversification: 8–6 Ma and 3–2.5 Ma [18]. The seven major lineages of *Tiquilia* (Boraginaceae), another diverse North American desert plant group, became differentiated ~ 23–13 Ma and experienced a marked increase in diversification beginning ~ 7 Ma [19]. Cacti (Cactaceae), the most spectacular New World succulent plants, originated ~ 35 Ma, but their species-rich clades originated in the late Miocene (~ 10–5 Ma) [20]. Central Asian *Reaumuria* (Tamaricaceae) began to diverge at the Eocene-Oligocene boundary and its two sections diversified in the early Miocene (22.5–19.8 Ma) [21]. In Australia, arid-adapted Camphorosmeae (Chenopodiaceae) became differentiated in the late Miocene (~ 7 Ma) [22]. These studies indicate that the timing of diversification of arid-adapted plant groups in different continents might have been inconsistent, but the period between the Miocene to the Pliocene was vital for the establishment of dryland floras globally. The majority of current studies focused on the evolutionary history of local arid-adapted plant groups, and did not investigate the evolution of dryland floras from a global view. Moreover, organismal capacities for adapting to novel ecological conditions can differ markedly [23]. To better understand the evolutionary processes that contributed to species richness in the different dryland floras, the diversification patterns of arid-adapted plant groups must be investigated in a broader phylogenetic context.

As an important representative in drylands [24, 25], Zygophyllaceae offers an opportunity for studying the diversification of dryland floras on a worldwide basis. This family consists of 22 genera and ~ 285 species, which are distributed throughout drylands of the world with a few extending to neighboring regions [25]. Zygophyllaceae plants are among the most important and characteristic components on the basis of their contribution to the flora and impact on the environment [25, 26]. For example, *Zygophyllum* and *Fagonia* are distributed widely in African dryland and are regarded as major components of the flora [27]; Creosotebush (*Larrea tridentata*) is one of the most dominant species in the New World dryland [24]. Morphological and anatomical features indicate that Zygophyllaceae plants can use water efficiently and are well adapted to dryland habitats [28, 29]. Additionally, Zygophyllaceae is one of 19 angiosperm families that use the C_4_ photosynthetic pathway [30], which is advantageous under the threat of extreme conditions (e.g. drought, sun, and high temperature) [31]. According to a large-scale molecular dating analysis in angiosperms, Zygophyllaceae originated in the early Paleocene [32].

In this study, we reconstruct a large-scale phylogenetic tree for Zygophyllaceae using four DNA markers with the most extensive taxon sampling to date. Within the large-scale phylogenetic framework, we then estimate the timing and patterns of lineage accumulation of Zygophyllaceae on global and regional scales. Finally, we investigate whether the rise of dryland floras in different continents were synchronous.

## Methods

### Molecular sequence data

One protein-coding gene (*rbcL*) and two noncoding regions (*trnL* intron and *trnL-F* intergenic spacer) of plastid DNA and one noncoding region, the internal transcribed spacers (ITS) of nuclear DNA were used for phylogenetic analysis. DNA sequences for all taxa were obtained from GenBank (www.ncbi.nlm.nih.gov). Accession numbers are listed in Additional file 1: Table S1. A total of 157 species from 21 of the 22 genera of Zygophyllaceae were sampled, with an additional 7 outgroup species from other rosids. Only the monotypic genus *Metharme* was not included because material was not available. At the species level, 55% of the currently 285 recognized species were included. The sequences of each marker were aligned and manually adjusted in BioEdit v.7.0.1 [33].

### Phylogenetic analysis

Phylogenetic analyses were initially performed for each marker using maximum likelihood (ML) method in RAxML [34]. The bootstrap support for conflicting nodes showed no significance among the individual markers (considered to exceed 70%), and the four individual datasets were therefore combined. Detailed analyses were carried out using ML and Bayesian inference (BI) methods. The best-fit model of nucleotide substitutions for each DNA region was determined by the Akaike information criterion in jModelTest v.2.1.4 [35], as follows: GTR + Γ for ITS and GTR + I + Γ for *rbcL*, *trnL*, and *trnL-F*. The parameters of the best-fit model for each DNA region are listed in Additional file 1: Table S2. RAxML was conducted using 1000 replicates with the fast bootstrap option. Bayesian analyses were carried out in MrBayes v.3.2.2 [36]. Two independent runs with each comprised four Markov Chain Monte Carlo (MCMC) chains (one cold and three heated) were conducted, starting from random trees. Chains were run for 30 million generations, sampling one tree every 1000 generations. The stationarity of the runs was assessed using Tracer v.1.6 [37]. A majority rule (> 50%) consensus tree was constructed after removing a burn-in of the initial 25% of the sampled trees, and the posterior probability was used to evaluate nodal robustness.

In order to assess the impact of missing data on phylogenetic reconstruction, we generated three additional matrixes of Zygophyllaceae: matrix 1 including 22 taxa, each taxon with four loci (22 taxa, 4 markers), matrix 2 (76 taxa, 3–4 markers), and matrix 3 (142, 2–4 markers). These three matrices were analysed in RAxML. The results indicated that the backbone of Zygophyllaceae generated from the dataset with 164 taxa (Additional file 2: Figure S1) is highly congruent with those obtained from the above three matrices (Additional file 2: Figure S2) except for nodes with poor support. The dataset with 164 taxa (each taxon with 1–4 markers) was therefore used in followed analyses.

### Divergence time estimates

Divergence times were estimated in BEAST v.1.8.1 [38], which employs a Bayesian MCMC approach to co-estimate topology and node ages. Dating analyses were conducted under the best-fit model for each marker partition, and a birth-death tree prior, with rate variation across branches uncorrelated and lognormal distributed. The MCMC chains were run for 50 million generations with parameters sampled every 1,000 generations. Tracer v.1.6 [37] was used to assess the adequate effective sample size (ESS) values (> 200) and the appropriate burn-in. The maximum clade credibility (MCC) tree with mean ages and 95% highest posterior density (HPD) intervals on nodes was generated using TreeAnnotator v1.8.0.

Phylogenetic position of Zygophyllales is poorly resolved within rosids [39]. The inclusion of sparse outgroups may violate a basic assumption of Bayesian dating approaches, namely even taxon sampling across lineages [40]. Therefore, rather than including rosid outgroups, we rooted the timetree using the mono-generic Krameriaceae (represented by *Krameria ixine* and *Krameria lanceolata*), sister to Zygophyllaceae [39]. The Zygophyllaceae fossil record is limited and cannot be placed confidently in the tree of extant taxa [41]. Moreover, no fossil has been reported for Krameriaceae. We used a secondary calibration point with normal prior distributions, 70 Ma (95% HPD: 49–88 Ma) for the split between Krameriaceae and Zygophyllaceae, obtained from a dense fossil-calibrated family-level phylogeny of angiosperms [32]. For comparison, we also used 60.9 Ma (34–90 Ma) recently estimated by Magallón et al. [42] to constrain the same node. Between these analyses, divergence times are highly congruent except that deep nodes vary less than *c.* 4%. The divergence times obtained using the age of the family-level timetree [32] as calibration was therefore reported and used for biogeographic and diversification analyses.

### Ancestral area reconstructions

Ancestral area states were reconstructed using Statistical Dispersal-Vicariance Analysis (S-DIVA) in the RASP software package [43]. We scored four main regions: A, Africa; B, Asia; C, Australia; and D, New World. In this analysis, the New World was not divided into North America and South America, because North American Zygophyllaceae species are clustered with South American species in our phylogeny, and the biotic interchange across the Americas occurred continually at ~ 20–6 Ma [44]. The S-DIVA analysis was performed using 1000 trees sampled randomly from the BEAST output as a “trees file”, and the MCC tree was used as a final representative tree.

### Diversification analyses

We implemented four methods to shed light on the temporal diversification patterns of Zygophyllaceae on global and regional scales. We employed Bayesian analysis of macro-evolutionary mixtures (BAMM), implemented in BAMM v.2.5 [45] to assess diversification rate heterogeneity along the branches. Incomplete taxon sampling was accounted for by assigning the percentage of species sampled (55%). We performed two independent runs on the MCC tree with a reversible jump MCMC run of 10 million generations, sampling parameters every 1000 generations. ESS values were computed in the R package CODA (> 200). The configuration of the diversification rate shifts was estimated using the posterior distribution, and Bayes factors were used to compare alternative diversification models. Results were analyzed and plotted using the R package BAMMtools v.2.0.2 [46]. Moore et al. [47] criticized this method and considered that the posterior on the number of shifts was overly sensitive to Poisson prior values. However, Rabosky et al. [48] demonstrated that the conclusion of Moore et al. [47] resulted from an invalid likelihood function, and suggest that in the most recent version of BAMM (v.2.5), the extreme sensitivity to the prior cannot be replicated. Considering this ongoing debate, we performed multiple BAMM analyses, similar to previous studies of *Campanula* [49] and Theaceae [11], by using various prior values (0.5, 1, 2, 5, and 10) to determine whether our results were affected by different prior settings.

We also used TreePar [50] to identify the locations of temporal shifts in global diversification rates of Zygophyllaceae. Similar to the way Couvreur et al. [51] examined the diversification rates of Arecaceae, we first determined the time point at which incomplete taxon sampling might begin to have a significant effect on TreePar analysis. Here, we found a dramatic increase of missing taxa occurred at 8.7 Ma (21% rising to 30%). TreePar analyses were run under the BD model with the following settings: start = 8.7, end = crown age of Zygophyllaceae, grid = 0.1 Ma. Rate shifts were determined using the likelihood ratio test (*p* < 0.05).

To visualize the accumulation of lineages within different regions, we fit exponential curves to the plots of Africa, Asia, Australia, and New World divergences drawn from the MCC tree and 100 trees sampled randomly from the post-burnin posterior distribution of the BEAST analysis as implemented by in-house R script (available upon request from the corresponding authors). In addition, we examined regional diversification of Zygophyllaceae using nodal “lineage density” method [52], which can reflect diversity due to both *situ* diversification and independent immigration events [53]. Geographic distribution at each node was obtained from S-DIVA analysis. Six species have two or multiple distribution states and were excluded from these two analyses.

## Results and discussion

### Phylogeny of Zygophyllaceae

Phylogenetic analyses of the combined four-marker datasets with different taxon and character sampling strategies yielded topologies (Additional file 2: Figures S1 and S2) that are highly congruent with those of previous studies [41, 54]. Zygophyllaceae and its five subfamilies (Zygophylloideae, Larreoideae, Seetzenioideae, Tribuloideae, and Morkillioideae) are all strongly supported as monophyletic. Within Zygophylloideae, six major clades are identified, which correspond to *Augea*, *Fagonia*, *Melocarpum*, *Roepera*, *Tetraena*, and *Zygophyllum* clades [41, 55]. Based on the geographic distribution, the *Roepera* clade can be divided further into two subclades: African subclade (I) and Australian subclade (II), nevertheless the supports for these two subclades are weak. Similarly, two major subclades (III and IV) are recognized in the *Fagonia* clade, distributed in Africa and the New World, respectively.

### Time and mode of diversification in Zygophyllaceae

**Fig. 1:**
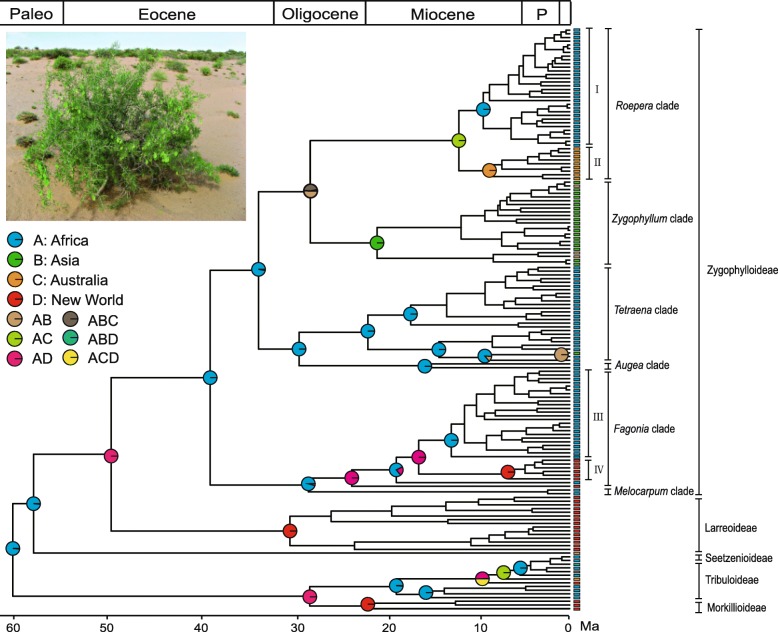
Chronogram of Zygophyllaceae with ancestral area reconstructions. Color-coded bars at the tips of the tree indicate the contemporary distribution of the corresponding species. Color-coded pie diagrams at each node show the relative probabilities of alternative ancestral distributions obtained by Statistical Dispersal-Vicariance Analysis (S-DIVA) optimizations over the 1000 trees from the BEAST analysis. An example of dryland flora dominated by *Zygophyllum xanthoxylum* is presented in the upper left. Photograph by S-X Yu

Divergence time estimates for Zygophyllaceae are shown in Additional file 2: Figure S3. Ancestral area reconstruction using the S-DIVA analysis is shown in Fig. 1. The crown group age of Zygophyllaceae is here estimated to be 59.89 Ma (95% HPD: 38.14–80.95 Ma), overlapping with the ages inferred by using *rbcL* data with two secondary calibrations and using ITS data with one secondary calibration [55]. The most recent common ancestor of the family occupied Africa, and multiple independent migrations from ancestral region to other dryland regions took place since the Eocene. Five dispersals into the New World occurred at ca. 49 Ma, 28 Ma, 23 Ma, 16 Ma, and 9 Ma, correspondingly generating Larreoideae, Morkilloideae, *Fagonia scoparia*, *Fagonia* subclade IV, and *Kallstroemia maxima*, respectively. Two dispersals into Asia generated *Zygophyllum* clade and *Tetraena mongolica* at ca. 28 Ma and 10 Ma, respectively. Two dispersals into Australia resulted in *Roepera* subclade II and *Tribulopis* at ca. 12 Ma and 7 Ma, respectively.

**Fig. 2:**
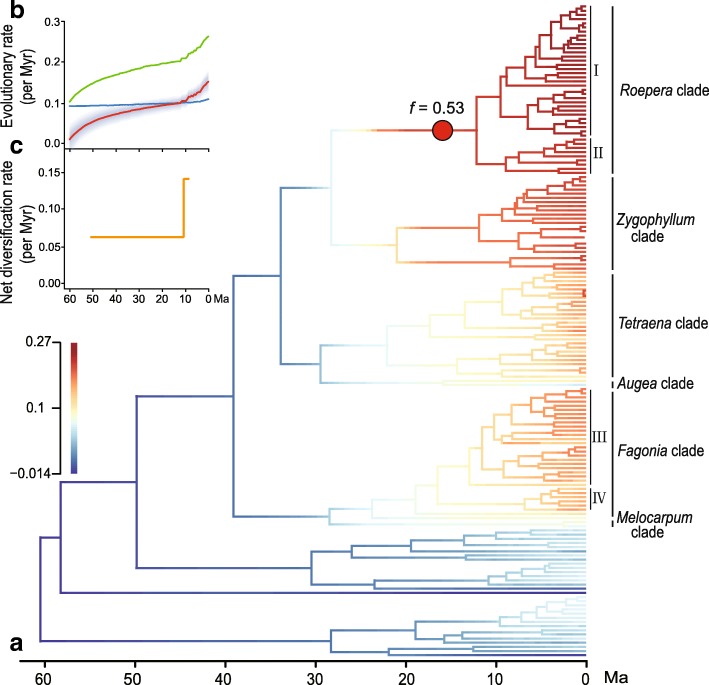
Diversification rates through time and among lineages during the evolutionary history of Zygophyllaceae. **a** Phylorate inferred from BAMM analysis under the prior of ‘ExpectedNumberofShifts’ as 1. Colors of branches denote directionality and strength of rate change, cooler and warmer colors designate slower and faster rates, respectively (see inset scale). The red circle indicates the position of shift in the maximum sampled *posterior* configuration. **b** Rate-through-time plots for rates of speciation (green), extinction (blue) and net species diversification (red, with grey probability distribution). **c** Maximum likelihood diversification rate estimates according to TreePar

Different tested Poisson priors (0.5, 1, 2, 5, and 10) from the BAMM analyses generated highly similar results. Following the suggestion of Rabosky et al. [45] for small trees (< 500 tips), we here described the results based on the analysis of prior 1 (Fig. 2). One acceleration of diversification rates was detected in the *Roepera* clade by sampling the maximum a posteriori configuration with the highest frequency (*f* = 0.53), ~ 15 Ma (Fig. 2a), and its net diversification rates are 0.23 species per million years (myr). BAMM analyses also indicate that the branch colors of six monophyletic groups, African *Roepera* (clade I), Australian *Roepera* (clade II), African *Tetraena* and *Fagonia* (clade III), and Asian *Zygophyllum*, and New World *Fagonia* (clade IV), became increasingly warm since the mid to late Miocene (Fig. 2a), displaying elevated rates of net diversification for each of these regional clades compared with the rest of the family (see inset scale in Fig. 2).

The rate-through-time plots suggest that the global speciation and net diversification rates of Zygophyllaceae significantly accelerated from about 10 Ma towards the present (Fig. 2b). TreePar analyses also rejected the null hypothesis of the constant diversification rate of the family (χ^2^ = 15.91, *p* = 0.0004), and found the one-shift model as the best. Net diversification rates of Zygophyllaceae on the whole increased from 0.053 species/myr to 0.142 species/myr at 10.4 Ma (Fig. 2c).

**Fig. 3:**
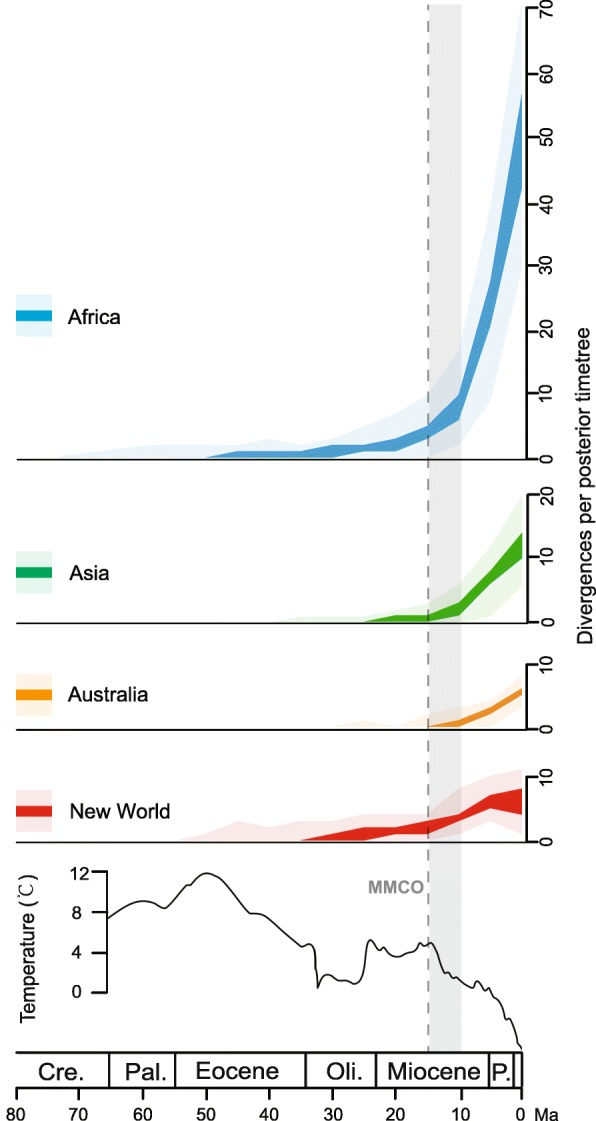
Zygophyllaceae divergences through time, according to geographic areas. Plots summarize the results of BEAST analyses of, and ancestral area reconstructions across, 101 posterior trees (see *Materials and Methods*). For each 5-million-year interval, the interquartile range (dark colors) and the complete span (light colors) of observed divergences are provided. The depiction of sea-surface temperature changes is modified from Zachos et al. [16]. Abbreviations: Cre. = Cretaceous; Pal. = Paleocene; Oli. = Oligocene; P. = Pliocene. Our results indicate that the initiation of diversification of Zygophyllaceae in different regions (light-grey shade) occurred after the MMCO (dashed line)

**Fig. 4:**
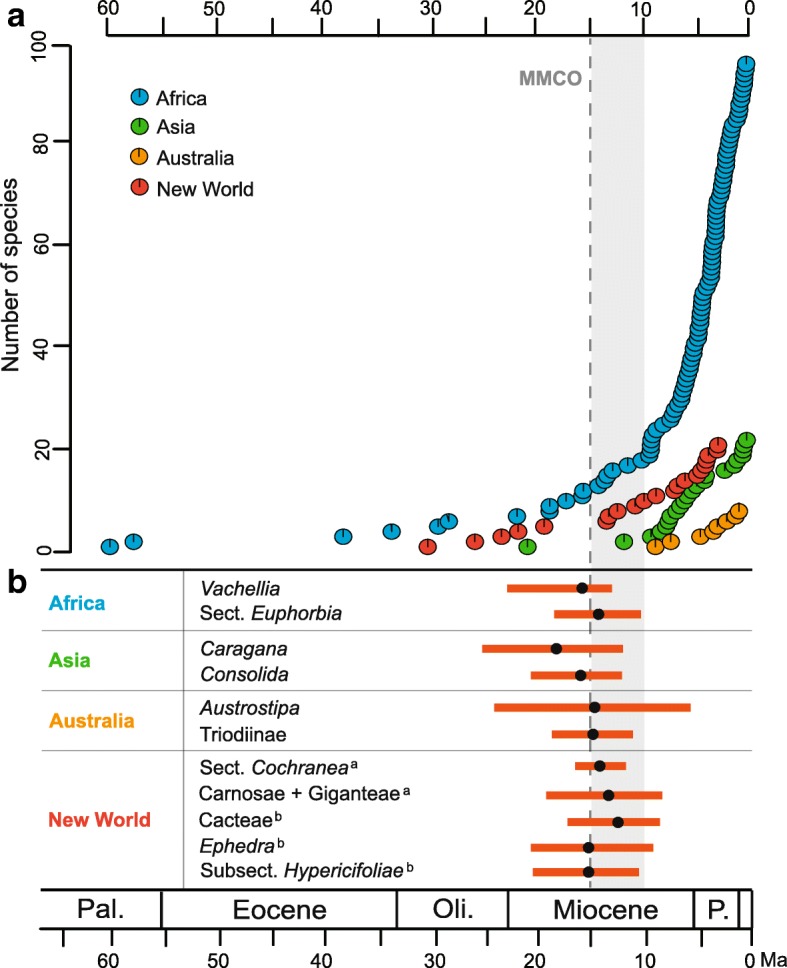
Diversification of plant groups in drylands. **a** Zygophyllaceae species accumulation estimates over time. Pie diagrams are color coded to reflect the proportion of alternative ancestral distributions obtained by the S-DIVA. **b** The timing of diversification for eleven representative plant groups in different dryland regions. The dark spots and their corresponding scale bars represent the mean ages and 95% HPD intervals (see *Discussion* for details). ^a, b^Taxa are from tropical America and western North America, respectively. Abbreviations: Pal. = Paleocene; Oli. = Oligocene; P. = Pliocene

The extant Zygophyllaceae dates to the Paleocene of Africa, ~ 60 Ma, but based on our region-specific divergence plots, the rapid accumulation of African lineages did not occur until the mid-Miocene (Figs. 3 and 4a). At the same time, lineages of Zygophyllaceae in Asia, Australia and the New World all experienced a dramatic increase after the mid-Miocene Climatic Optimum (MMCO, ~ 15 Ma; Figs. 3 and 4a).

### Diversification burst of the global dryland floras

Our analyses show that extant Zygophyllaceae date to the Paleocene of Africa, and migrations from ancestral areas to other regions did not take place synchronously. However, rapid accumulations of Zygophyllaceae lineages in four dryland regions (Africa, Asia, Australia, and the New World) occurred relatively recently, beginning at the mid-late Miocene (~ 15–10 Ma). The timing of these major diversification events within Zygophyllaceae is similar to those of other representative plant groups in different dryland regions, such as African *Vachellia* (Fabaceae) and Sect. *Euphorbia* (Euphorbiaceae), Asian *Caragana* (Fabaceae) and *Consolida* (Ranunculaceae), Australian *Austrostipa* (Poaceae) and Triodiinae (Poaceae), tropical American Sect. *Cochranea* (Heliotropiaceae) and Carnosae + Giganteae (Oxalidaceae), and western North American Cacteae (Cactaceae), *Ephedra* (Ephedraceae) and Subsect. *Hypericifoliae* (Euphorbiaceae) (Fig. 4b; Additional file 1: Table S3). Moreover, a rapid accumulation of spiny plant lineages, as well as mammalian herbivores in African savanna, occurred since the mid-Miocene [56]. Australia *Acacia* (Fabaceae) underwent an accelerated diversification at ~ 15 Ma [57]. The New World Cactaceae had a significant shift in diversification rates at 16.0–14.8 Ma [20]. In western North America, the ivesioids clade of *Potentilla* (Rosaceae) experienced a westward range expansion to the Sierra Nevada and the coast of California 12 Ma [58]. Thus, we suggest that the establishment of modern dryland floras in different continents was nearly synchronous, beginning in the mid-late Miocene.

The near-synchronous initiation of rise of dryland floras across the multiple continents indicates that a global trigger may have been responsible. After the MMCO, a steep and steady decline in global temperatures occurred (Fig. 3; [16, 59]), which possibly resulted in a decrease of global precipitation on account of a slowdown of the hydrological cycle [20]. Lines of evidence from paleoecology [60] and stable isotopes [61, 62] from multiple continents robustly support a general trend toward increasing aridity from the MMCO onwards. Several studies have suggested that global climate cooling was the main driver contributing to the long-term drying trend since the mid-Miocene and shaped the modern flora [63–66]. Thus, it is possible that the global rise of dryland floras was largely driven by the global climate change that reduced global precipitation and thereby led to a global expansion of arid environments.

The rise of dryland floras in different continents might have also been linked to the enhanced aridification and expansion of drylands due to regional orogenetic and climate changes. Uplift of the East African Plateau occurred during the early Miocene ~ 17 Ma, which resulted in the formation of aridity in east Africa [67, 68]. With the shrinkage of the Paratethys Sea during the late Miocene, the African monsoon was enhanced drastically, generating arid conditions across North Africa [69]. Broad-scale uplift of plateaus and mountains occurred in the northern and eastern Tibetan Plateau, the east-central Andes and Altiplano, the east African rift valley, and the northern Canadian Rockies in the last 15 Ma [6, 70–73]. Atmosphere and biosphere simulations indicate that the uplift itself might have led to a drastic reorganization of atmospheric circulation, engendering strong aridification and climate changes [74, 75]. A significant advance of Antarctic ice occurred at ~ 14 Ma [76], and at the same time, sea levels began to fall across southeastern Australia, eventually retreating towards the current coastline at ~ 10–9 Ma, heralding the onset of different conditions across continental Australia [77].

## Conclusions

In summary, the present study reveals that the rise of dryland floras across multiple continents is near-synchronous, beginning at the mid-late Miocene. The causative factors for the global near-synchronous rise were suggested to be a band of a global climate cooling; however, regional orogenetic and climate changes may have played additional important roles. Our findings highlight the mid-late Miocene as an essential period for the assembly and evolution of modern dryland floras, although the original timings of dryland floras in different continents might have been asynchronous. Yet, Zygophyllaceae is only one of many components of dryland floras. This hypothesis needs to be further tested by studying other plant groups of dryland floras in a broad phylogenetic context.

## Additional files

Additional file 1: **Table S1.** Species, voucher information and GenBank accession numbers for the dataset of four markers. **Table S2.** The parameters of the best-fit model for each DNA region. **Table S3.** The crown group ages and ancestral habitat types of eleven representative plant groups in different drylands. (PDF 143 kb)

Additional file 2: **Figure S1.** Phylogenetic relationships of Zygophyllaceae based on the 4-marker dataset with 164 taxa. **Figure S2.** Phylogenetic relationships of Zygophyllaceae based on three matrices (see Methods for details). **Figure S3.** Chronogram of Zygophyllaceae obtained from the BEAST analysis of the four-marker dataset. **Figure S4.** Ancestral habitat reconstructions for five representative plant groups inhabiting in both dryland and non-dryland floras. (PDF 2101 kb)

## Abbreviations

BAMM: Bayesian analysis of macro-evolutionary mixtures
BI: Bayesian inference
HPD: Highest posterior density
ITS: Internal transcribed spacers
Ma: Million years ago
MCC: Maximum clade credibility
MCMC: Markov Chain Monte Carlo
ML: Maximum likelihood
MMCO: Mid-Miocene Climatic Optimum
myr: Million years
S-DIVA: Statistical Dispersal-Vicariance Analysis
