# Supplementary material for: Insights into the historical assembly of global dryland floras: the diversification of Zygophyllaceae
Source: BMC Evol Biol. 2018 Nov 9;18:166. doi: 10.1186/s12862-018-1277-z (PMC6234786; doi:10.1186/s12862-018-1277-z)
Supplement: Supplementary file 1 — Table S1. Species, voucher information and GenBank accession numbers for the dataset of four markers. Table S2. The parameters of the best-fit model for each DNA region. Table S3. The crown group ages and ancestral habitat types of eleven representative plant groups in different drylands. (PDF 143 kb) [file 12862_2018_1277_MOESM1_ESM.pdf]

Additional file 1

Table S1 Species, voucher information and GenBank accession numbers for the datasets of four markers.

Table S2 The parameters of the best-fit model for each DNA region.

Table S3 The crown group ages and ancestral habitat types of eleven representative plant groups in dryland floras across different continents.

Table S1

Species	Voucher	*rbcL*	*trnL*	*trnL-F*	ITS
Zygophyllum album L.f.	Thulin *et al.* 7977 (UPS)	AJ133861	AJ387963	AJ387963	
Zygophyllum aff. maritimum Dold	Dold 4654 (GRA)		EF656034	EF656034	
Zygophyllum apiculata F. Muell.	Greder 18664 (K)		AY233384		
Zygophyllum applanatum Van Zyl	Bellstedt 870 (STE)		EF656012	EF656012	
Zygophyllum atriplicoides Fisch. & Mey.	Astanova s.n. (K)		AY233385		
Zygophyllum aurantiacum (Lindl.) F. Muell.	Greder 20900 (K)		AY300775		
Zygophyllum billardierei DC.	R. 417 (Adelaide B.G.)	AJ133862	AJ387964	AJ387964	
Zygophyllum botulifolium Van Zyl	Marais 451 (STE)		EF656026	EF656026	
Zygophyllum brachypterum Kar. & Kir.	H65 (KUN)[1]; XJBIZLJ021 (XJBI)[2]	JF944800[1]	KR001987[2]	KR002014[2]	KR002024[2]
Zygophyllum calcicola Van Zyl	Dreyer s.n. (STE)		EF656030	EF656030	
Zygophyllum chrysopteron Retief	Marais 427 (STE)		EF656013	EF656013	
Zygophyllum clavatum Schltr. & Diels	Bellstedt 878 (STE)	EF655986	EF656010	EF656010	
Zygophyllum coccineum L.	Ryding 1347 (K)	AJ133863	AJ387965	AJ387965	
Zygophyllum compressa J. M. Black	Nicholls 809 (K)		AY300776		
Zygophyllum cordifolium L. f.	Marais 446 (STE)	EF655993	EF656022	EF656022	
Zygophyllum cretaceum Van Zyl	Bellstedt 856 (STE)		EF656028	EF656028	
Zygophyllum cuneifolium Eckl. & Zeyh.	Marais 455 (STE)		EF656024	EF656024	
Zygophyllum cylindrifolium Schinz	Craven 3800 (WIND)	AJ133864	AJ387966	AJ387966	
Zygophyllum debile Cham.	Bellstedt 796 (STE)		EF656041	EF656041	
Zygophyllum decumbens Delile	Thulin *et al.* 7981 (UPS)	AJ133865	AJ387967	AJ387967	
Zygophyllum divaricatum Eckl. & Zeyh.	Dold 4655 (GRA)		EF656031	EF656031	
Zygophyllum eremaea Diels	Beier s.n. (UPS)		AY300777		
Zygophyllum fabago L.	Chase 516 (K)[1]; XJBIZLJ025 (XJBI)[2]	Y15030[1]	KR001984[2]	KR002011[2]	KR002029[2]
Zygophyllum flexuosum Eckl. & Zeyh.	Bellstedt 794 (STE)	EF655995	EF656032	EF656032	
Zygophyllum foetidum Schrad. & J. C. Wendl.	Marais 423 (STE)		EF656039	EF656039	
Zygophyllum fruticulosum DC.	Chase 2203 (K)	AJ133866	AJ387969	AJ387969	
Zygophyllum fulvum L.	Van Zyl 4605 (STE)		EF656044	EF656044	
Zygophyllum fuscatum Van Zyl	Bellstedt 892 (STE)		EF656045	EF656045	
Zygophyllum fusiforme Van Zyl	Bellstedt 857 (STE)		EF656023	EF656023	
Zygophyllum giessii Merxm. & A. Schreib.	Bellstedt 874 (STE)	EF655980	EF656000	EF656000	
Zygophyllum glaucum F. Muell.	Chase 2204 (K)	AJ133867	AJ387970	AJ387970	
Zygophyllum gobicum Maxim.	XJBIZLJ022 (XJBI)		KR001995	KR002008	KR002031
Zygophyllum gontscharovii Boriss.	Astanova s.n. (K)		AY300787		
Zygophyllum hamiense Schweinf.	Thulin *et al.* 9840 (UPS)		AY300783		
Zygophyllum hildebrandtii Engl.	Thulin *et al.* 9012 (UPS)	AJ133868	AJ387971	AJ387971	
Zygophyllum hirticaule Van Zyl	Van Zyl 3894 (STE)	AJ133869	AJ387973	AJ387973	
Zygophyllum iliense Popov	XJBIZLJ023 (XJBI)		KR001993	KR002006	KR002032
Zygophyllum incrustatum E. Mey. ex	Bellstedt 509 (STE)		EF656019	EF656019	

Sond.					
Zygophyllum inflatum Van Zyl	HK 1490 (WIND)		EF656005	EF656005	
Zygophyllum iodocarpum F. Muell.	Symon 4607 (K)		AY300779		
Zygophyllum jaxarticum Popov	XJBIZLJ026 (XJBI)				KR002034
Zygophyllum kansuense Y. X. Liou	XJBIZLJ024 (XJBI)		KR001996	KR002010	KR002022
Zygophyllum kaschgaricum Boriss.	XJBIZLJ012 (XJBI)		KR001981	KR002000	KR002018
Zygophyllum lehmannianum Bunge	June s.n. 1972 (K)		AY300788		
Zygophyllum leptopetalum E. Mey. ex Sond.	Marais 422 (STE)		EF656040	EF656040	
Zygophyllum leucocladum Diels	Van Zyl 4479 (STE)		EF656029	EF656029	
Zygophyllum lichtensteinianum Cham.	Van Zyl 4594 (STE)		EF656020	EF656020	
Zygophyllum loczyi Kanitz	XJBIZLJ019 (XJBI)		KR001988	KR002015	KR002021
Zygophyllum longicapsulare Schinz	Bellstedt 879 (STE)	EF655981	EF656001	EF656001	
Zygophyllum macropodum Boriss.	D1508 (KUN)[1]; XJBIZLJ001 (XJBI)[2]	JF944806[1]			KR002030[2]
Zygophyllum macropterum C. A. Mey.	XJBIZLJ017 (XJBI)		KR001991	KR002003	KR002026
Zygophyllum maculatum Aiton	Marais 433 (STE)		EF656033	EF656033	
Zygophyllum madagascariensis (Baill.) Stauffer	Keating Miller 2236 (K)		AY300784		
Zygophyllum madecassum H. Perrier	Lorence s.n. (K)		AY300785		
Zygophyllum maritimum Eckl. & Zeyh.	Dold 4656 (GRA)		EF656035	EF656035	
Zygophyllum microcarpum E. Mey.	Van Zyl 4591 (STE)	EF655983	EF656002	EF656002	
Zygophyllum migiurtinorum Chiov.	Thulin *et al.* 9553 (UPS)		AY300786		
Zygophyllum miniatum Cham.	June s.n. 1965 (K)		AY300789		
Zygophyllum morgsana L.	Bellstedt 890 (STE)	EF655994	EF656021	EF656021	
Zygophyllum mucronatum Maxim.	XJBIZLJ030 (XJBI)		KR001997	KR002009	KR002023
Zygophyllum namaquanum Van Zyl	Marais 440 (STE)		EF656036	EF656036	
Zygophyllum obliquum Popov	H67 (KUN)[1]; XJBIZLJ028 (XJBI)[2]	JF944808[1]	KR001989[2]	KR002002[2]	KR002028[2]
Zygophyllum orbiculatum Welw. ex Oliv.	Craven 5096 (WIND)	EF655979	EF655999	EF655999	
Zygophyllum ovatum Ewart & J. White	Melville 451 (K)		AY300782		
Zygophyllum oxycarpum Popov	XJBIZLJ031 (XJBI)		KR001992	KR002007	KR002033
Zygophyllum patenticaule Van Zyl	Bellstedt 868 (STE)	EF655989	EF656008	EF656008	
Zygophyllum porphyrocaule Van Zyl	Bellstedt 800 (STE)	EF655992	EF656018	EF656018	
Zygophyllum potaninii Maxim.	XJBIZLJ020 (XJBI)		KR001986	KR002013	KR002020
Zygophyllum prismatocarpum Sond.	Bellstedt 860 (STE)	EF655990	EF656009	EF656009	
Zygophyllum pterocarpum Bunge	H64 (KUN)[1]; XJBIZLJ016 (XJBI)[2]	JF944809[1]	KR001985[2]	KR002012[2]	KR002025[2]
Zygophyllum pterocaule Van Zyl	Mucina 270806/25 (STE)		EF656007	EF656007	
Zygophyllum pubescens Schinz	Bellstedt 881 (STE)		EF656042	EF656042	
Zygophyllum pygmaeum Eckl. & Zeyh.	Marais 424 (STE)		EF656046	EF656046	
Zygophyllum ramosissimum Popov	Granitov s.n. (K)		AY300790		
Zygophyllum retrofractum Thunb.	Marais 430 (STE)		EF656014	EF656014	
Zygophyllum rigidum Schinz	Van Zyl 4590 (STE)	EF655982	EF656003	EF656003	
Zygophyllum robecchii Engl.	Chase 636 (K)	AJ133870	AJ387972	AJ387972	

Zygophyllum rogersii Compton	Marais 432 (STE)		EF656037	EF656037	
Zygophyllum rosowii Bunge	H62 (KUN)[1]; XJBIZLJ027 (XJBI)[2]	JF944811[1]	KR001994[2]	KR002005[2]	KR002035[2]
Zygophyllum schreiberanum Merxm. & Giess	Bellstedt 871 (STE)		EF656027	EF656027	
Zygophyllum segmentatum Van Zyl	Bellstedt 861 (STE)	EF655987	EF656015	EF656015	
Zygophyllum sessilifolium L.	Marais 434 (STE)	EF655997	EF656047	EF656047	
Zygophyllum simplex L.	Chase 806 (K)	Y15031	AJ387974	AJ387974	
Zygophyllum spinosum L.	Bellstedt 801 (STE)	AJ133871	EF656038	EF656038	
Zygophyllum spitskopense Van Zyl	Van Zyl 4606 (STE)		EF656048	EF656048	
Zygophyllum spongiosum Van Zyl	HK 1573 (WIND)	EF655985	EF656006	EF656006	
Zygophyllum subtrijugum C. A. Mey.	1955.07.30 s. leg. s.n. (K)		AY300792		
Zygophyllum swartbergense Van Zyl	Bellstedt 798 (STE)	EF655996	EF656043	EF656043	
Zygophyllum tenue R. Glover	Van Zyl 4593 (STE)		EF656017	EF656017	
Zygophyllum teretifolium Schltr.	Marais 447 (STE)		EF656025	EF656025	
Zygophyllum turbinatum Van Zyl	Bellstedt 799 (STE)		EF656016	EF656016	
Zygophyllum xanthoxylum (Bunge) Maxim.	Chase 1700 (K)[1]; XJBIZLJ013 (XJBI)[2]	AJ133872[1]	KR001982[2]	KR002001[2]	KR002019[2]
Fagonia acerosa Boiss.	Davis 56261 (E)		AY641579		AY641617
Fagonia arabica L.	Leonard 4887 (S)		AY641580		AY641618
Fagonia bruguieri DC.	Thulin *et al.* 9986 (UPS)		AY641582		AY641619
Fagonia charoides Chiov.	Thulin *et al.* 10587 (UPS)		AY641583		
Fagonia chilensis Hook. & Arn.	Penailillo s.n. (UTALCA)		AY641584		AY641622
Fagonia cretica L.	Chase 3432 (K)[1]	AJ133855[1]	AJ387942[1]	AJ387942[1]	
Fagonia densa I. M. Johnst.	Rebman 3171 (SD)		AY641587		AY641625
Fagonia glutinosa Delile	Davis 49654 (K)		AY641588		AY641627
Fagonia gypsophila Beier & Thulin	Thulin *et al.* 9473 (UPS)		AY641589		AY641626
Fagonia hadramautica Beier & Thulin	Thulin *et al.* 9808 (UPS)		AY641590		AY641628
Fagonia harpago Emb. & Maire	Podlech 40630 (RSA)		AY641591		AY641629
Fagonia indica Burm. f.	Thulin *et al.* 9835 (UPS)	Y15018	AY300769	AJ387943	AY641630
Fagonia laevis Standl.	Beier 97 (UPS)		AY641595		AY641633
Fagonia lahovarii Volkens & Schweinf.	Thulin *et al.* 9522 (UPS)		AY641596		AY641635
Fagonia latifolia Delile	Scholz 174 (B)		AY641597		
Fagonia latistipulata Beier & Thulin	Thulin *et al.* 10833 (UPS)		AY641598		AY641636
Fagonia longispina Batt.	Podlech 53369 (M)		AY641599		AY641637
Fagonia luntii Baker	Thulin *et al.* 9881 (UPS)	AJ133856	AJ387944	AJ387944	AY641638
Fagonia mahrana Beier	Thulin *et al.* 9682 (UPS)		AY641600		AY641639
Fagonia minutistipula Engl.	Giess and Müller 13952 (K)		AY300771		AY641641
Fagonia mollis Delile	Townsend 86/12 (K)		AY641601		
Fagonia olivieri DC.	Samuelsson 4357 (S)		AY641602		AY641646
Fagonia orientalis C. Presl	Collenette 7516 (E)		AY641603		AY641648
Fagonia pachyacantha Rydb.	Beier 93 (UPS)		AY641604		AY641649
Fagonia palmeri Vasey & Rose	Hastings 75 (SD)		AY641605		AY641653
Fagonia paulayana J. Wagner & Vierh.	Thulin *et al.* 9515 (UPS)		AY641608		AY641654

Fagonia rangei Loes. ex Engl.	Leistner 3388 (K)		AY641609		AY641647
Fagonia scabra Forssk.	Davis 49662 (E)		AY300768		
Fagonia scoparia Brandegee	Johnston 9461 (SD)		AY300773		AY641644
Fagonia subinermis Boiss.	Grey-Wilson & Hewer 285 (W)		AY641610		AY641642
Fagonia villosa D. M. Porter	K. 5915 (RSA)		AY641611		AY641640
Fagonia zilloides Humbert	Davis 49047 (E)		AY641612		AY641655
Augea capensis Thunb.	Bellstedt 934 (STE)	EF655978	EF655998		
Tetraena mongolica Maxim.	Sheahan 1994 (K)[1]; XJBIZLJ015 (XJBI)[2]	Y15027[1]	KR001983[2]	KR001999[2]	KR002017[2]
Bulnesia arborea (Jacq.) DC.	Chase 641 (K)	EU644676	AJ387947	AJ387947	
Guaiacum angustifolium Engelm.	J. R. Dertien 534 (DEK)		EU253465	EU253465	JX486127
Guaiacum coulteri A. Gray	L. Lopez & M. Martinez 196 (DEK)		EU253466	EU253466	JX486715
Guaiacum guatemalense Planch. ex Rydb. & Vail	Chase 640 (K)	Y15019	AJ387948	AJ387948	JX486717
Guaiacum officinale L.	J.R. Dertien 505 (DEK)		EU253467	EU253467	JX901024
Guaiacum sanctum L.	L. Lopez & M. Martinez 204 (DEK)	JQ594515	EU253458	EU253461	JX486719
Guaiacum unijugum Brandegee	R. McCauley s.n. (DEK)		JX682629	JX682629	JX486721
Porlieria chilensis I.M. Johnst	Chase 643 (K)	Y15024	AJ387955	AJ387955	JX901026
Larrea ameghinoi Speg.	J.H.H.13246 (SI)	AF333329			AF334825
Larrea cuneifolia Cav.	CUNE	AF200471			JF267296
Larrea divaricata Cav.	J.H.H.9886 (SI)	AF200472			AF334821
Larrea nitida Cav.	J.H.H.13240 (SI)	AF200473			AF334823
Larrea tridentata (Sessé & Moc. ex DC.) Coville	Chase 636 (K)[1]; R. Laport 766242 (RSA)[2]	AY935748	AJ387951[1]	AJ387951[1]	JF267306[2]
Pintoa chilensis Gay	Teillier 859 (NY)	AJ133858	AJ387954	AJ387954	
Plectrocarpa tetracantha Gillies ex Hook. & Arn.	J. H. Hunziker and V. Lia 13269	AF333330			
Seetzenia lanata (Willd.) Bullock	Herman 3964 (K)	Y15025	AJ387956	AJ387956	
Tribulopis pentandra R.Br.	Wilson 4719 (NSW)	AJ133860	AJ387960	AJ387960	
Tribulus cistoides L.	J.R. Abbott 24857 (FLAS)	GU135188			
Tribulus lanuginosus L.	Sathishkumar *et al.* (2010, unpublished)				HM236860
Tribulus macropterus Boiss	Collenette 3/93 (K)	Y15028	AJ387961	AJ387961	
Tribulus subramanyamii P. Singh, G.S. Giri & V. Singh	Sathishkumar *et al.* (2010, unpublished)				HM236858
Tribulus terrestris L.	XJBIZLJ014 (XJBI)	AM235167	KR001980	KR001998	KR002016
Kallstroemia maxima (L.) Hook. & Arn.	Magellanes 3806 (K)	Y15020	AJ387949	AJ387949	
Kelleronia revoilii	Barbier s.n. (K)	Y15021	AJ387950	AJ387950	
Sisyndite spartea E.Mey. ex Sond.	Chase 637 (K)	Y15026	AJ387958	AJ387958	
Neoluederitzia sericeocarpa Schinz	Chase 642 (K)	Y15023	AJ387953	AJ387953	
Balanites maughamii Sprague	Chase 635 (K)	JF265296	AJ387946	AJ387946	
Balanites pedicellaris Mildbr. & Schltr.	OM901	JF265297			
Morkillia mexicana (DC.) Rose & Painter	Anderson 13526 (MICH, NY)	AJ133857	AJ387952	AJ387952	
Viscainoa geniculata (Kellogg) Greene	Chase 634 (K)	Y15029	AJ387962	AJ387962	

Sericodes greggii A. Gray	Rollins & Roby 76014 (NY, H)	AJ387962	AJ387957	AJ387957	
Krameria lanceolata Torr.	Simpson 88-05-1-1 (MICH)	Y15032			AY261080
Krameria ixine L.	Fernandez 22529	EU644679			AY260995
Albizia procera (Roxb.) Benth.	DS14502_JM1604	KC417044	EU440009		
Geranium sibiricum L.	Seonjoo and Woo 2040501	JX913498	DQ267175		
Sterculia pruriens (Aubl.) K. Schum.	NH200020	JQ625993	FJ039232		
Punica granatum L.	Hao 2000-0318 (SYS)	JQ730672	AY905483		
Vitis aestivalis Michx.	Wen 10428 (US)	L01960	HM223286		

Table S2

Dataset	Model	-ln*L*	AIC	A: C: G: T	P_inv	Shape
rbcL	GTR + I + Γ	6793.5049	13863.0098	0.2850: 0.1787: 0.2250: 0.3113	0.5900	0.9210
trnL	GTR + I + Γ	5361.7718	11347.5437	0.4338: 0.1174: 0.1321: 0.3167	0.1970	1.5150
trnL-F	GTR + I + Γ	3342.2175	7120.435	0.4642: 0.1291: 0.1133: 0.3934	0.0000	2.2380
ITS	GTR + Γ	7770.0399	15826.0797	0.2392: 0.2956: 0.2637: 0.2014	-	0.6150

The best-fit model was determined by the Akaike information criterion (AIC) in jModelTest. “-” indicates inapplicable.

Table S3

Region	Taxon	Crown group age (95% HPD)	Ancestral habitat	Comment
**Africa**				
	Vachellia (Fabaceae)	16.6 (13.5–23.4)	dryland	African species of *Vachellia* is mainly distributed in dry woodlands except that three species inhabit in mesic forests [1]. Our inference of habitat evolution indicates that the most recent common ancestor (MRCA) of African *Vachellia* occurred in dryland habitat (Additional file 2: Figure S4a).
	Sect. *Euphorbia* (Euphorbiaceae)	14 (10.5–17.8)	dryland	Most of Sect. *Euphorbia* species are dominants of dryland floras throughout Africa (including Madagascar) except that a few of species inhabit in humid montane regions [2, 3]. Our inference of habitat evolution indicates that the MRCA of Sect. *Euphorbia* lived in dryland habitat (Additional file 2: Figure S4b).
**Asia**				
	Caragana (Fabaceae)	18.37 (12.11–25.87)	dryland (steppe)	Species of *Caragana* are primarily restricted to dryland floras, with a few outliers extending to non-dryland floras. Zhang et al. inferred evolution of habitats in the genus and found that its MRCA inhabited in Junggar steppe of Northwest dryland region of China [4].
	Consolida (Ranunculaceae)	16.9 (13.4–20.9)	dryland	Species of *Consolida* are mainly distributed in central Asian and Irano-Turanian dryland regions, with a few extending to the Mediterranean basin [5]. Our inference of habitat evolution indicates that the MRCA of *Consolida* occurred in dryland habitat (Additional file 2: Figure S4c).
**Australia**				
	Austrostipa (Poaceae)	14.55 (6.48–24.21)	dryland	Genus *Austrostipa* is a major component of dryland grasslands in Australia, with a few outliers extending to other habitats [6]. Our inference of habitat evolution indicates that the MRCA of *Consolida* occurred in dryland habitat (Additional file 2: Figure S4d).
	Triodiinae (Poaceae)	14.7 (11.4–18.3)	dryland (eremaean)	Toon et al. reconstruct evolution of habitats in Triodiinae and identified eremaean as the ancestral biome for the subtribe [7].
**New World**				
	Sect. *Cochranea* (Heliotropiaceae)[a]	14 (12–16)	dryland	Based on Luebert and Wen [8], Sect. *Cochran*ea is entirely distributed in dryland regions.
	Carnosae + Giganteae (Oxalidaceae)[a]	13.29 (7.81–19.19)	dryland (desert)	Heibl and Renner inferred evolution of habitats of *Oxalis* and found that Carnosae and Giganteae of the genus diversified in desert [9].
	Cacteae (Cactaceae)[b]	11.94 (8.33–17.27)	dryland (desert)	Tribe Cacteae is mainly distributed in the southern Chihuahuan Desert [10].

			Hernández-Hernández et al. performed the reconstruction of ancestral ranges in Cactaceae and indicated that the MRCA of the tribe occurred in the southern Chihuahuan Desert [11].
Ephedra (Ephedraceae)[b]	15.31 (9.84–20.89)	dryland	Based on Lorea et al. [12], western North American *Ephedra* plants are entirely restricted to in dryland regions.
Subsect. *Hypericifoliae* (Euphorbiaceae)[b]	15.3 (10.5–20.2)	dryland	Subsect. *Hypericifoliae* is mainly distributed in dryland regions of North America, with a few species growing in the understory of mesic forests [3]. Our inference of habitat evolution indicates that the MRCA of the subsection occurred in dryland habitat (Additional file 2: Figure S4e).

HPD = highest posterior density. [a,b]Taxa are from tropical America and western North America, respectively.

Notes: Five plant groups, *Vachellia*, Sect. *Euphorbia*, *Consolida*, *Austrostipa*, and Subsect. *Hypericifoliae*, are primarily restricted to dryland habitats, with a few outliers extending to non-dryland habitats. In order to determine their ancestral habitats, we inferred evolutionary patterns of habitats using parsimony method as implemented in Mesquite v2.75 [13]. To simplify our analyses, habitat types were coded as dryland or non-dryland. Data are obtained from several sources (Electronic databases: www.efloras.org; personal observation in herbarium specimens; and GBIF website (https://www.gbif.org/)). The phylogenetic trees of these five groups are modified from the trees generated from previous studies: African *Vachellia* from Bouchenak-Khelladi et al. [1], Sect. *Euphorbia* and Subsect. *Hypericifoliae* from Horn et al. [3], *Consolida* from Jabbour and Renner [5], and *Austrostipa* from Syme [6]. Our inferring results are indicated in Additional file 2: Figure S4a-e. For *Caragana*, Triodiinae, and Cacteae, which occur in dryland and nor-dryland habitats, Zhang et al. [4], Toon et al. [7], and Hernández-Hernández et al. [11] have ascertained dryland habitats as their ancestral floras, respectively. Other three groups are entirely distributed in dryland floras: Sect. *Cochranea* [8], Carnosae + Giganteae [9], and North American *Ephedra* [12].

References

1. Bouchenak-Khelladi Y, Maurin O, Hurter J, van der Bank M. The evolutionary history and biogeography of Mimosoideae (Leguminosae): An emphasis on African acacias. Mol Phylogenet Evol. 2010;57:495–508.
2. Horn JW, van Ee BW, Morawetz JJ, Riina R, Steinmann VW, Berry PE, et al. Phylogenetics and the evolution of major structural characters in the giant genus *Euphorbia* L. (Euphorbiaceae).Mol Phylogenet Evol. 2012;63:305–26.
3. Horn JW, Xi ZX, Riina R, Peirson JA, Yang Y, Dorsey BL, et al. Evolutionary bursts in *Euphorbia* (Euphorbiaceae) are linked with photosynthetic pathway. Evolution. 2014;68:3485–504.
4. Zhang ML, Xiang XG, Xue JJ, Sanderson SC, Fritsch PW. Himalayan uplift shaped biomes in Miocene temperate Asia: evidence from leguminous *Caragana*. Sci Rep. 2016;6:36528.
5. Jabbour F, Renner SS. *Consolida* and *Aconitell*a are an annual clade of *Delphinium* (Ranunculaceae) that diversified in the Mediterranean basin and the Irano-Turanian region. Taxon. 2011;60:1029–40.
6. Syme AE. Diversification rates in the Australasian endemic grass *Austrostipa*: 15 million years of constant evolution. Plant Syst Evol. 2012;298:221–7.
7. Toon A, Crisp MD, Gamage H, Mant J, Morris DC, Schmidt S, et al. Key innovation or adaptive change? A test of leaf traits using Triodiinae in Australia. Sci Rep. 2015;5:12398.
8. Luebert F, Wen J. Phylogenetic Analysis and Evolutionary Diversification of Heliotropium Sect. *Cochranea* (Heliotropiaceae) in the Atacama Desert. Syst Bot. 2008;33:390–402.
9. Heibl C, Renner SS. Distribution Models and a Dated Phylogeny for Chilean *Oxalis* Species Reveal Occupation of New Habitats by Different Lineages, not Rapid Adaptive Radiation. Syst Biol. 2012;61:823–34.
10. Hernández HM, Gómez-Hinostrosa C. Cactus diversity and endemism in the Chihuahuan Desert Region. In: Cartron JL, Editor. Biodiversity and conservation in Northern Mexico. Oxford University Press; 2005. pp. 264–275.
11. Hernández-Hernández T, Brown JW, Schlumpberger BO, Eguiarte LE, Magallón S. Beyond aridification: multiple explanations for the elevated diversification of cacti in the New World Succulent Biome. New Phytol. 2014;202:1382–97.
12. Loera I, Sosa V, Ickert-Bond S. Diversification in North American arid lands: Niche conservatism, divergence and expansion of habitat explain speciation in the genus *Ephedra*. Mol Phylogenet Evol. 2012;65:437–50.
13. Maddison WP, Maddison DR. 2011. Mesquite: a modular system for evolutionary analysis, v1.12. Available at http://mesquiteproject.org. Accessed 21 Aug 2017.
