# Supplementary material for: Insights into the historical assembly of global dryland floras: the diversification of Zygophyllaceae
Source: BMC Evol Biol. 2018 Nov 9;18:166. doi: 10.1186/s12862-018-1277-z (PMC6234786; doi:10.1186/s12862-018-1277-z)
Supplement: Supplementary file 2 — Figure S1. Phylogenetic relationships of Zygophyllaceae based on the 4-marker dataset with 164 taxa. Figure S2. Phylogenetic relationships of Zygophyllaceae based on three matrices (see Methods for details). Figure S3. Chronogram of Zygophyllaceae obtained from the BEAST analysis of the four-marker dataset. Figure S4. Ancestral habitat reconstructions for five representative plant groups inhabiting in both dryland and non-dryland floras. (PDF 2101 kb) [file 12862_2018_1277_MOESM2_ESM.pdf]

Additional file 2

Figure S1 Phylogenetic relationships of Zygophyllaceae based on the 4-marker dataset with 164 taxa. The bootstrap values (> 50%) are shown above the branches, and the Bayesian posterior probabilities (> 0.90) are indicated below the branches. ML phylogram is presented in the upper left.

Figure S2 Phylogenetic relationships of Zygophyllaceae based on three matrices (see Methods for details). The bootstrap values (> 50%) are shown above the branches. (a) matrix 1 with 22 taxa, each taxa with four sequences; (b) matrix 2 with 76 taxa, each taxa with three to four sequences; (c) matrix 3 with 142 taxa, each taxa with two to four sequences.

Figure S3 Chronogram of Zygophyllaceae obtained from the BEAST analysis of the 4-marker dataset. Bars around the node ages indicate the 95% highest posterior density (HPD) intervals.

Figure S4 Ancestral habitat reconstructions for five representative plant groups inhabiting in both dryland and non-dryland floras. (a) Afrian *Vachellia* (Fabaceae); (b) Sect. *Euphorbia* (Euphorbiaceae); (c) *Consolida* (Ranunculaceae); (d) *Austrostipa* (Poaceae); and (e) Subsect. *Hypericifoliae* (Euphorbiaceae).

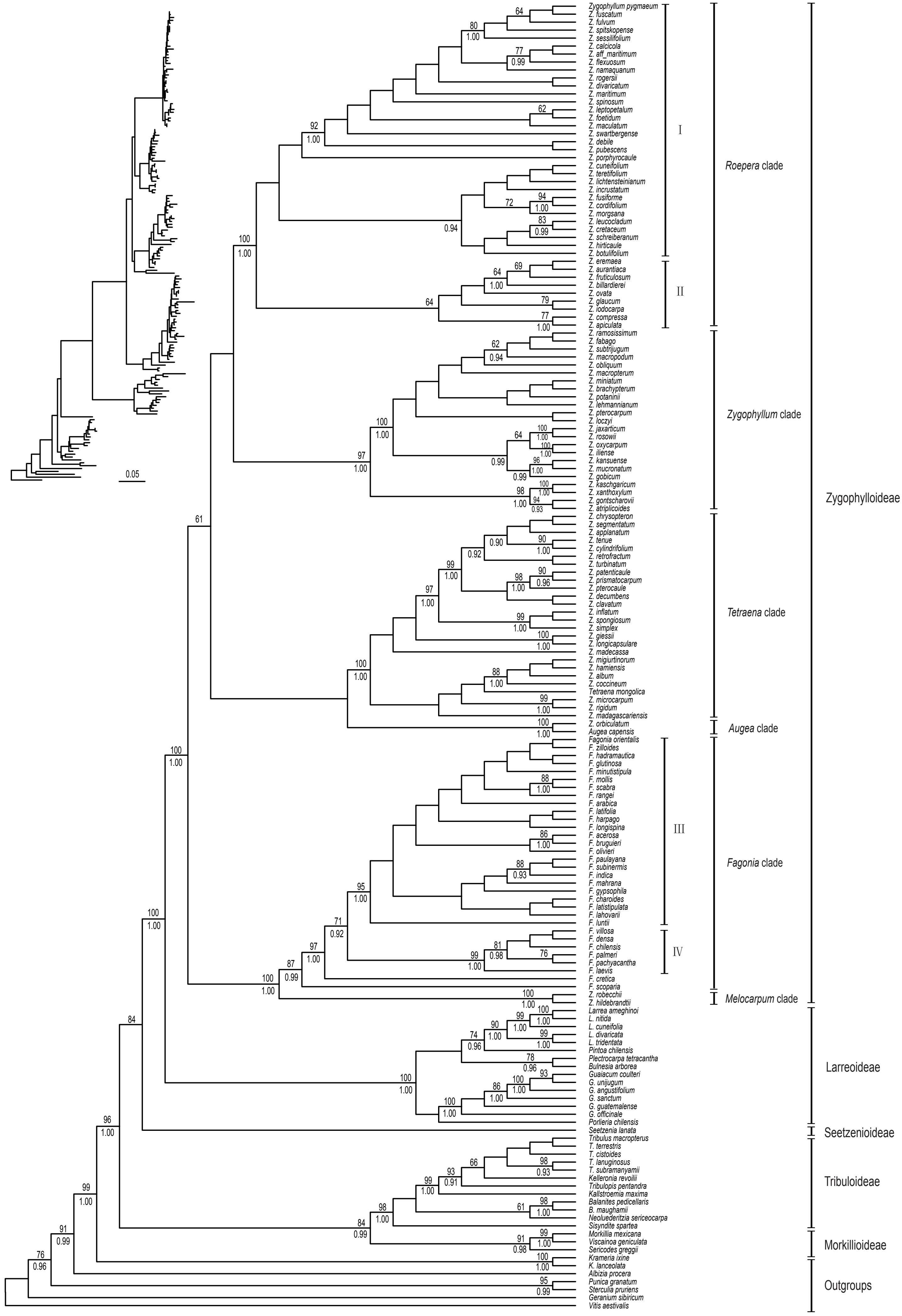
0.05
Zygophyllum pygmaeum
Z. fuscatum
Z. fulvum
Z. spitskopense
Z. sessilifolium
Z. calcicola
Z. aff_maritimum
Z. flexuosum
Z. namaquanum
Z. rogersii
Z. divaricatum
Z. maritimum
Z. spinosum
Z. leptopetalum
Z. foetidum
Z. maculatum
Z. swartbergense
Z. debile
Z. pubescens
Z. porphyrocaule
Z. cuneifolium
Z. teretifolium
Z. lichtensteinianum
Z. incrustatum
Z. fusiforme
Z. cordifolium
Z. morgsana
Z. leucocladum
Z. cretaceum
Z. schreiberanum
Z. hirticaule
Z. botulifolium
Z. eremaea
Z. aurantiaca
Z. fruticulosum
Z. billardierei
Z. ovata
Z. glaucum
Z. iodocarpa
Z. compressa
Z. apiculata
Z. ramosissimum
Z. fabago
Z. subtrijugum
Z. macropodum
Z. obliquum
Z. macropterum
Z. miniatum
Z. brachypterum
Z. potaninii
Z. lehmannianum
Z. pterocarpum
Z. loczyi
Z. jaxarticum
Z. rosowii
Z. oxycarpum
Z. iliense
Z. kansuense
Z. mucronatum
Z. gobicum
Z. kaschgaricum
Z. xanthoxylum
Z. gontscharovii
Z. atriplicoides
Z. chrysopteron
Z. segmentatum
Z. applanatum
Z. tenue
Z. cylindrifolium
Z. retrofractum
Z. turbinatum
Z. patenticaule
Z. prismatocarpum
Z. pterocaule
Z. decumbens
Z. clavatum
Z. inflatum
Z. spongiosum
Z. simplex
Z. giessii
Z. longicapsulare
Z. madecassa
Z. migiurtinorum
Z. hamiensis
Z. album
Z. coccineum
Tetraena mongolica
Z. microcarpum
Z. rigidum
Z. madagascariensis
Z. orbiculatum
Augea capensis
Fagonia orientalis
F. zilloides
F. hadramautica
F. glutinosa
F. minutistipula
F. mollis
F. scabra
F. rangei
F. arabica
F. latifolia
F. harpago
F. longispina
F. acerosa
F. bruguieri
F. olivieri
F. paulayana
F. subinermis
F. indica
F. mahrana
F. gypsophila
F. charoides
F. latistipulata
F. lahovarii
F. luntii
F. villosa
F. densa
F. chilensis
F. palmeri
F. pachyacantha
F. laevis
F. cretica
F. scoparia
Z. robecchii
Z. hildebrandtii
Larrea ameghinoi
L. nitida
L. cuneifolia
L. divaricata
L. tridentata
Pintoa chilensis
Plectrocarpa tetracantha
Bulnesia arborea
Guaiacum coulteri
G. unijugum
G. angustifolium
G. sanctum
G. guatemalense
G. officinale
Porlieria chilensis
Seetzenia lanata
Tribulus macropterus
T. terrestris
T. cistoides
T. lanuginosus
T. subramanyamii
Kelleronia revoilii
Tribulopis pentandra
Kallstroemia maxima
Balanites pedicellaris
B. maughamii
Neoluederitzia sericeocarpa
Sisyndite spartea
Morkillia mexicana
Viscainoa geniculata
Sericodes greggii
Krameria ixine
K. lanceolata
Albizia procera
Punica granatum
Sterculia pruriens
Geranium sibiricum
Vitis aestivalis
I
II
III
IV
Roepera clade
Zygophyllum clade
Tetraena clade
Augea clade
Fagonia clade
Melocarpum clade
Zygophylloideae
Larreoideae
Seetzenioideae
Tribuloideae
Morkillioideae
Outgroups

a.

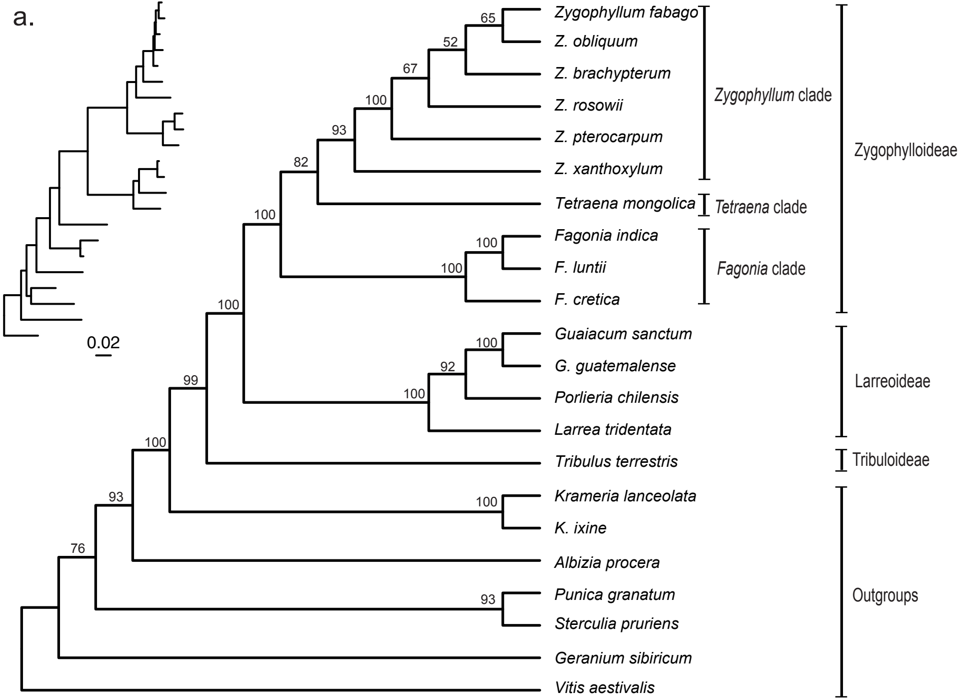

0.02
65
52
67
100
93
82
100
100
100
100
92
100
99
100
100
93
93
76
Zygophyllum fabago
Z. obliquum
Z. brachypterum
Z. rosowii
Z. pterocarpum
Z. xanthoxylum
Tetraena mongolica
Fagonia indica
F. luntii
F. cretica
Guaiacum sanctum
G. guatemalense
Porlieria chilensis
Larrea tridentata
Tribulus terrestris
Krameria lanceolata
K. ixine
Albizia procera
Punica granatum
Sterculia pruriens
Geranium sibiricum
Vitis aestivalis
Zygophyllum clade
Tetraena clade
Fagonia clade
Zygophylloideae
Larreoideae
Tribuloideae
Outgroups

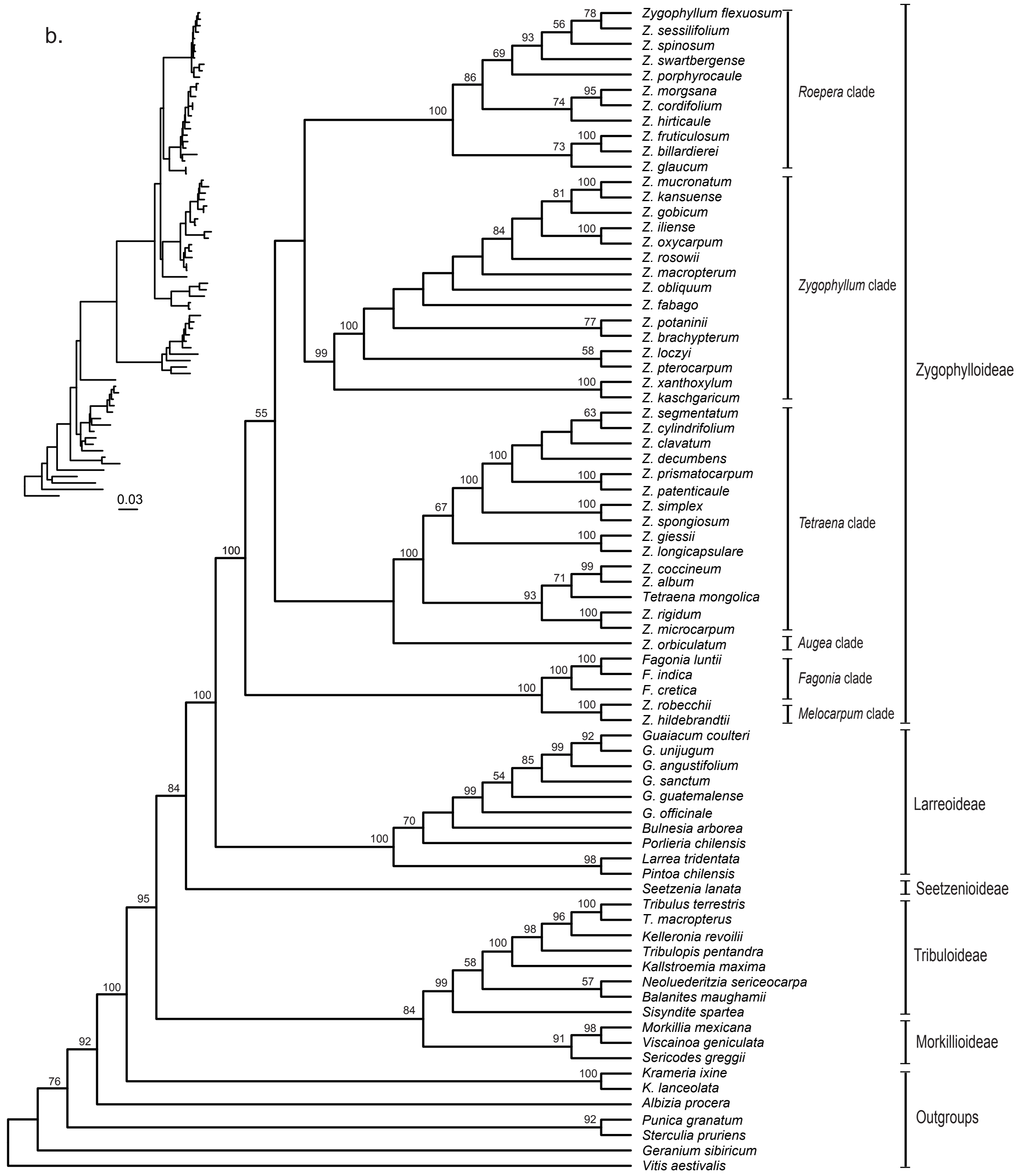
b.
0.03
Zygophyllum flexuosum
Z. sessilifolium
Z. spinosum
Z. swartbergense
Z. porphyrocaule
Z. morgsana
Z. cordifolium
Z. hirticaule
Z. fruticulosum
Z. billardierei
Z. glaucum
Z. mucronatum
Z. kansuense
Z. gobicum
Z. iliense
Z. oxycarpum
Z. rosowii
Z. macropterum
Z. obliquum
Z. fabago
Z. potaninii
Z. brachypterum
Z. loczyi
Z. pterocarpum
Z. xanthoxylum
Z. kaschgaricum
Z. segmentatum
Z. cylindrifolium
Z. clavatum
Z. decumbens
Z. prismatocarpum
Z. patenticaule
Z. simplex
Z. spongiosum
Z. giessii
Z. longicapsulare
Z. coccineum
Z. album
Tetraena mongolica
Z. rigidum
Z. microcarpum
Z. orbiculatum
Fagonia luntii
F. indica
F. cretica
Z. robecchii
Z. hildebrandtii
Guaiacum coulteri
G. unijugum
G. angustifolium
G. sanctum
G. guatemalense
G. officinale
Bulnesia arborea
Porlieria chilensis
Larrea tridentata
Pintoa chilensis
Seetzenia lanata
Tribulus terrestris
T. macropterus
Kelleronia revoilii
Tribulopis pentandra
Kallstroemia maxima
Neoluederitzia sericeocarpa
Balanites maughamii
Sisyndite spartea
Morkillia mexicana
Viscainoa geniculata
Sericodes greggii
Krameria ixine
K. lanceolata
Albizia procera
Punica granatum
Sterculia pruriens
Geranium sibiricum
Vitis aestivalis
Roepera clade
Zygophyllum clade
Tetraena clade
Augea clade
Fagonia clade
Melocarpum clade
Zygophylloideae
Larreoideae
Seetzenioideae
Tribuloideae
Morkillioideae
Outgroups

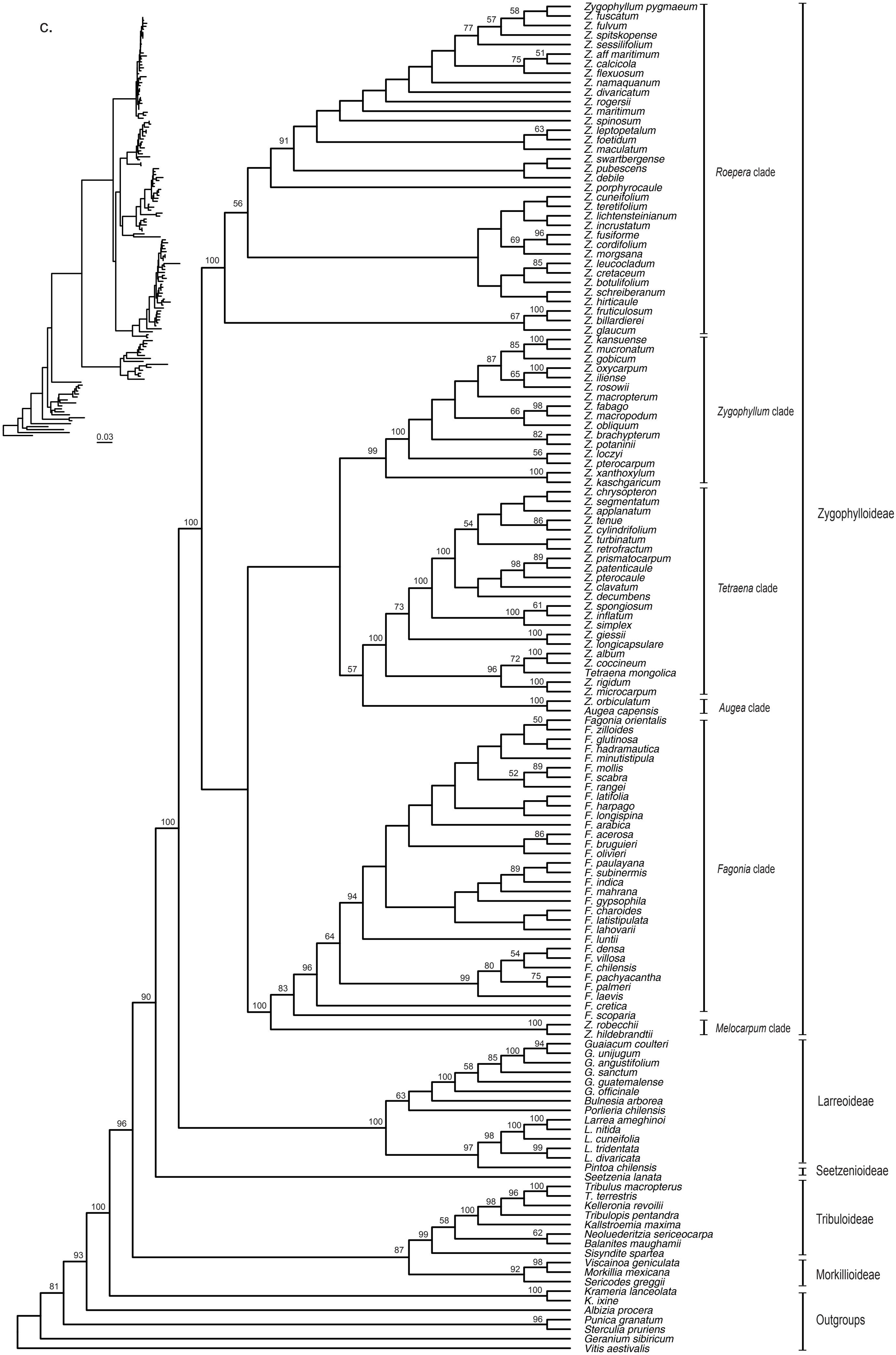

c.
0.03
Zygophyllum pygmaeum
Z. fuscatum
Z. fulvum
Z. spitskopense
Z. sessilifolium
Z. aff maritimum
Z. calcicola
Z. flexuosum
Z. namaquanum
Z. divaricatum
Z. rogersii
Z. maritimum
Z. spinosum
Z. leptopetalum
Z. foetidum
Z. maculatum
Z. swartbergense
Z. pubescens
Z. debile
Z. porphyrocaule
Z. cuneifolium
Z. teretifolium
Z. lichtensteinianum
Z. incrustatum
Z. fusiforme
Z. cordifolium
Z. morgsana
Z. leucocladum
Z. cretaceum
Z. botulifolium
Z. schreiberanum
Z. hirticaule
Z. fruticulosum
Z. billardierei
Z. glaucum
Z. kansuense
Z. mucronatum
Z. gobicum
Z. oxycarpum
Z. iliense
Z. rosowii
Z. macropterum
Z. fabago
Z. macropodum
Z. obliquum
Z. brachypterum
Z. potaninii
Z. loczyi
Z. pterocarpum
Z. xanthoxylum
Z. kaschgaricum
Z. chrysopteron
Z. segmentatum
Z. applanatum
Z. tenue
Z. cylindrifolium
Z. turbinatum
Z. retrofractum
Z. prismatocarpum
Z. patenticaule
Z. pterocaule
Z. clavatum
Z. decumbens
Z. spongiosum
Z. inflatum
Z. simplex
Z. giessii
Z. longicapsulare
Z. album
Z. coccineum
Tetraena mongolica
Z. rigidum
Z. microcarpum
Z. orbiculatum
Augea capensis
Fagonia orientalis
F. zilloides
F. glutinosa
F. hadramautica
F. minutistipula
F. mollis
F. scabra
F. rangei
F. latifolia
F. harpago
F. longispina
F. arabica
F. acerosa
F. bruguieri
F. olivieri
F. paulayana
F. subinermis
F. indica
F. mahrana
F. gypsophila
F. charoides
F. latistipulata
F. lahovarii
F. luntii
F. densa
F. villosa
F. chilensis
F. pachyacantha
F. palmeri
F. laevis
F. cretica
F. scoparia
Z. robecchii
Z. hildebrandtii
Guaiacum coulteri
G. unijugum
G. angustifolium
G. sanctum
G. guatemalense
G. officinale
Bulnesia arborea
Porlieria chilensis
Larrea ameghinoi
L. nitida
L. cuneifolia
L. tridentata
L. divaricata
Pintoa chilensis
Seetzenia lanata
Tribulus macropterus
T. terrestris
Kelleronia revoilii
Tribulopis pentandra
Kallstroemia maxima
Neoluederitzia sericeocarpa
Balanites maughamii
Sisyndite spartea
Viscainoa geniculata
Morkillia mexicana
Sericodes greggii
Krameria lanceolata
K. ixine
Albizia procera
Punica granatum
Sterculia pruriens
Geranium sibiricum
Vitis aestivalis
Roepera clade
Zygophyllum clade
Tetraena clade
Augea clade
Fagonia clade
Melocarpum clade
Zygophylloideae
Larreoideae
Seetzenioideae
Tribuloideae
Morkillioideae
Outgroups

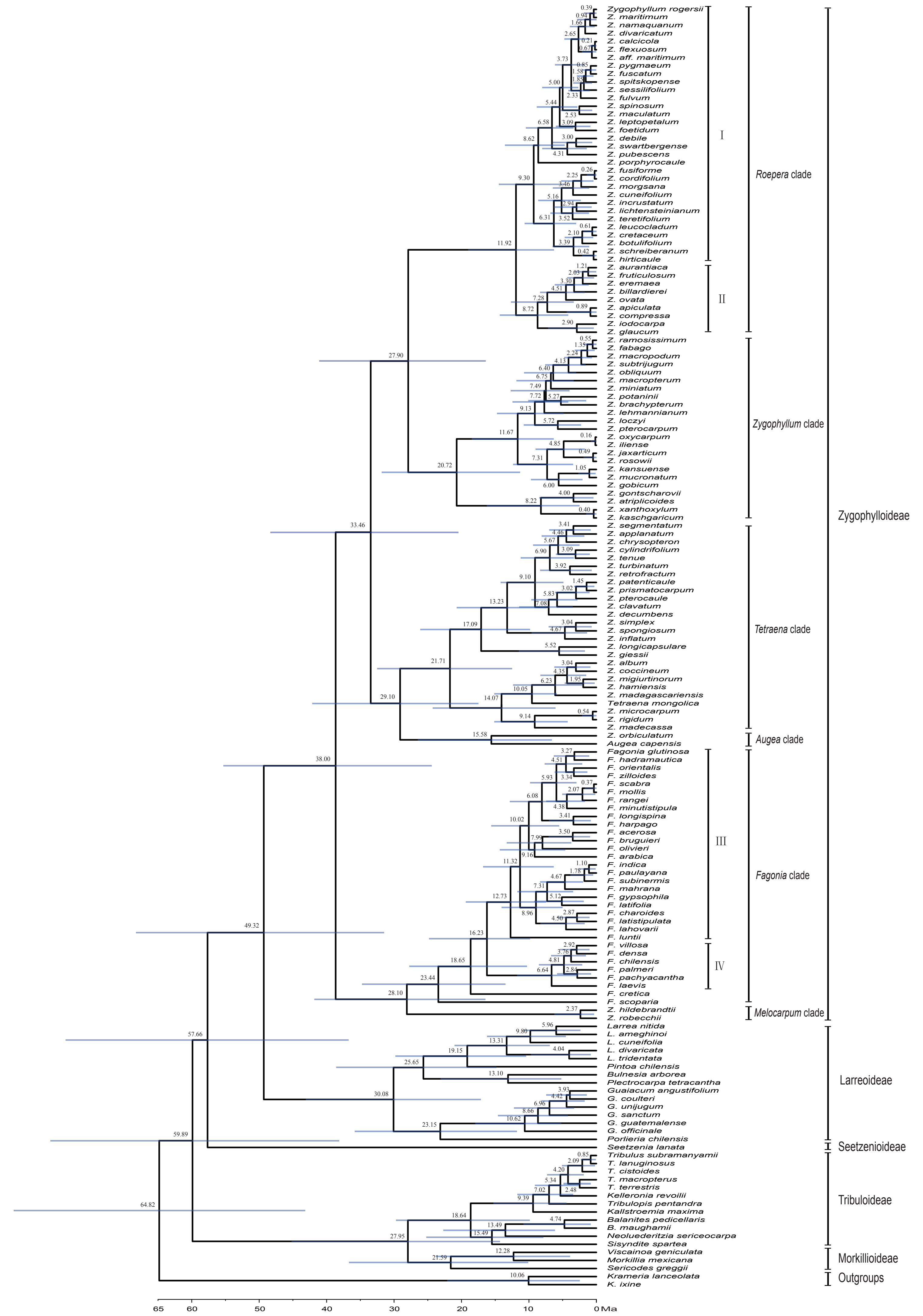

Zygophyllum rogersii
Z. maritimum
Z. namaquanum
Z. divaricatum
Z. calcicola
Z. flexuosum
Z. aff. maritimum
Z. pygmaeum
Z. fuscatum
Z. spitskopense
Z. sessilifolium
Z. fulvum
Z. spinosum
Z. maculatum
Z. leptopetalum
Z. foetidum
Z. debile
Z. swartbergense
Z. pubescens
Z. porphyrocaule
Z. fusiforme
Z. cordifolium
Z. morgsana
Z. cuneifolium
Z. incrustatum
Z. lichtensteinianum
Z. teretifolium
Z. leucocladum
Z. cretaceum
Z. botulifolium
Z. schreiberanum
Z. hirticaule
Z. aurantiaca
Z. fruticulosum
Z. eremaea
Z. billardierei
Z. ovata
Z. apiculata
Z. compressa
Z. iodocarpa
Z. glaucum
Z. ramosissimum
Z. fabago
Z. macropodum
Z. subtrijugum
Z. obliquum
Z. macropterum
Z. miniatum
Z. potaninii
Z. brachypterum
Z. lehmannianum
Z. loczyi
Z. pterocarpum
Z. oxycarpum
Z. iliense
Z. jaxarticum
Z. rosowii
Z. kansuense
Z. mucronatum
Z. gobicum
Z. gontscharovii
Z. atriplicoides
Z. xanthoxylum
Z. kaschgaricum
Z. segmentatum
Z. applanatum
Z. chrysopteron
Z. cylindrifolium
Z. tenue
Z. turbinatum
Z. retrofractum
Z. patenticaule
Z. prismatocarpum
Z. pterocaule
Z. clavatum
Z. decumbens
Z. simplex
Z. spongiosum
Z. inflatum
Z. longicapsulare
Z. giessii
Z. album
Z. coccineum
Z. migiurtinorum
Z. hamiensis
Z. madagascariensis
Tetraena mongolica
Z. microcarpum
Z. rigidum
Z. madecassa
Z. orbiculatum
Augea capensis
Fagonia glutinosa
F. hadramautica
F. orientalis
F. zilloides
F. scabra
F. mollis
F. rangei
F. minutistipula
F. longispina
F. harpago
F. acerosa
F. bruguieri
F. olivieri
F. arabica
F. indica
F. paulayana
F. subinermis
F. mahrana
F. gypsophila
F. latifolia
F. charoides
F. latistipulata
F. lahovarii
F. luntii
F. villosa
F. densa
F. chilensis
F. palmeri
F. pachyacantha
F. laevis
F. cretica
F. scoparia
Z. hildebrandtii
Z. robecchii
Larrea nitida
L. ameghinoi
L. cuneifolia
L. divaricata
L. tridentata
Pintoa chilensis
Bulnesia arborea
Plectrocarpa tetracantha
Guaiacum angustifolium
G. coulteri
G. unijugum
G. sanctum
G. guatemalense
G. officinale
Porlieria chilensis
Seetzenia lanata
Tribulus subramanyamii
T. lanuginosus
T. cistoides
T. macropterus
T. terrestris
Kelleronia revoilii
Tribulopis pentandra
Kallstroemia maxima
Balanites pedicellaris
B. maughamii
Neoluederitzia sericeocarpa
Sisyndite spartea
Viscainoa geniculata
Morkillia mexicana
Sericodes greggii
Krameria lanceolata
K. ixine
I
II
III
IV
Roepera clade
Zygophyllum clade
Tetraena clade
Augea clade
Fagonia clade
Melocarpum clade
Zygophylloideae
Larreoideae
Seetzenioideae
Tribuloideae
Morkillioideae
Outgroups
65
60
50
40
30
20
10
0 Ma

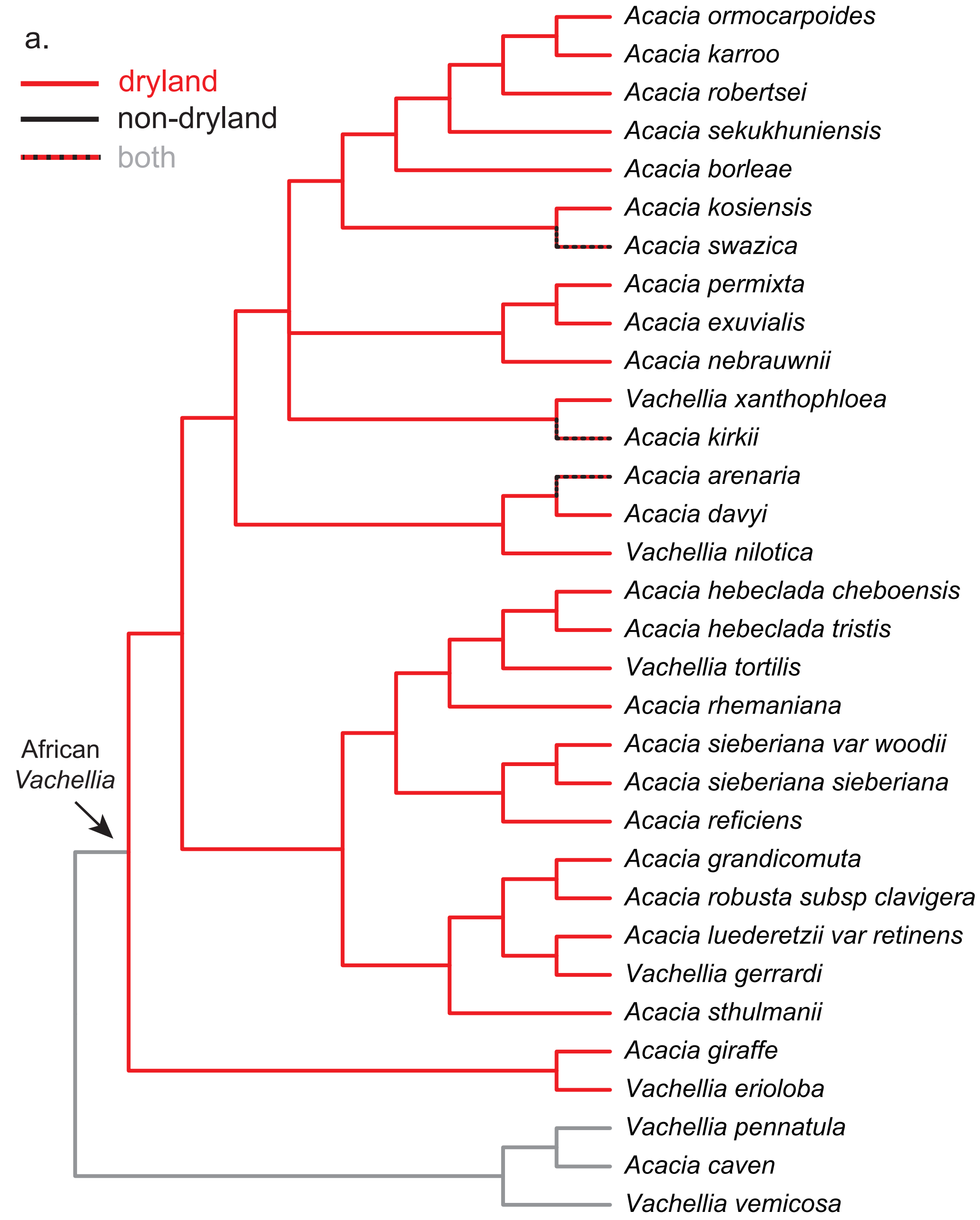

a.
dryland
non-dryland
both
African
Vachellia
Acacia ormocarpoides
Acacia karroo
Acacia robertsei
Acacia sekukhuniensis
Acacia borleae
Acacia kosiensis
Acacia swazica
Acacia permixta
Acacia exuvialis
Acacia nebrauwnii
Vachellia xanthophloea
Acacia kirkii
Acacia arenaria
Acacia davyi
Vachellia nilotica
Acacia hebeclada cheboensis
Acacia hebeclada tristis
Vachellia tortilis
Acacia rhemaniana
Acacia sieberiana var woodii
Acacia sieberiana sieberiana
Acacia reficiens
Acacia grandicomuta
Acacia robusta subsp clavigera
Acacia luederetzii var retinens
Vachellia gerrardi
Acacia sthulmanii
Acacia giraffe
Vachellia erioloba
Vachellia pennatula
Acacia caven
Vachellia vemicosa

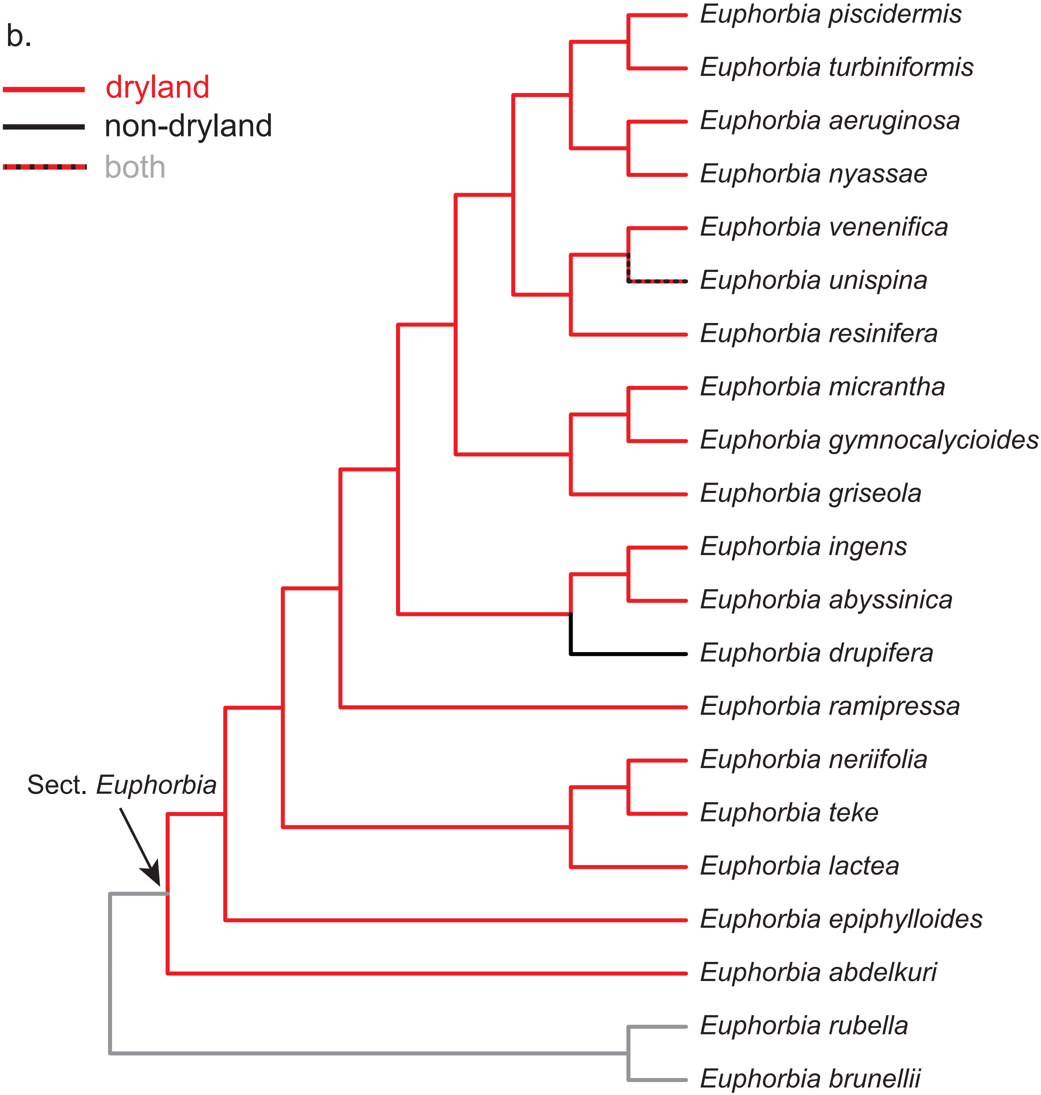
b.
dryland
non-dryland
both
Euphorbia piscidermis
Euphorbia turbiniformis
Euphorbia aeruginosa
Euphorbia nyassae
Euphorbia venenifica
Euphorbia unispina
Euphorbia resinifera
Euphorbia micrantha
Euphorbia gymnocalycioides
Euphorbia griseola
Euphorbia ingens
Euphorbia abyssinica
Euphorbia drupifera
Euphorbia ramipressa
Euphorbia neriifolia
Euphorbia teke
Euphorbia lactea
Euphorbia epiphylloides
Euphorbia abdelkuri
Euphorbia rubella
Euphorbia brunellii
Sect. Euphorbia

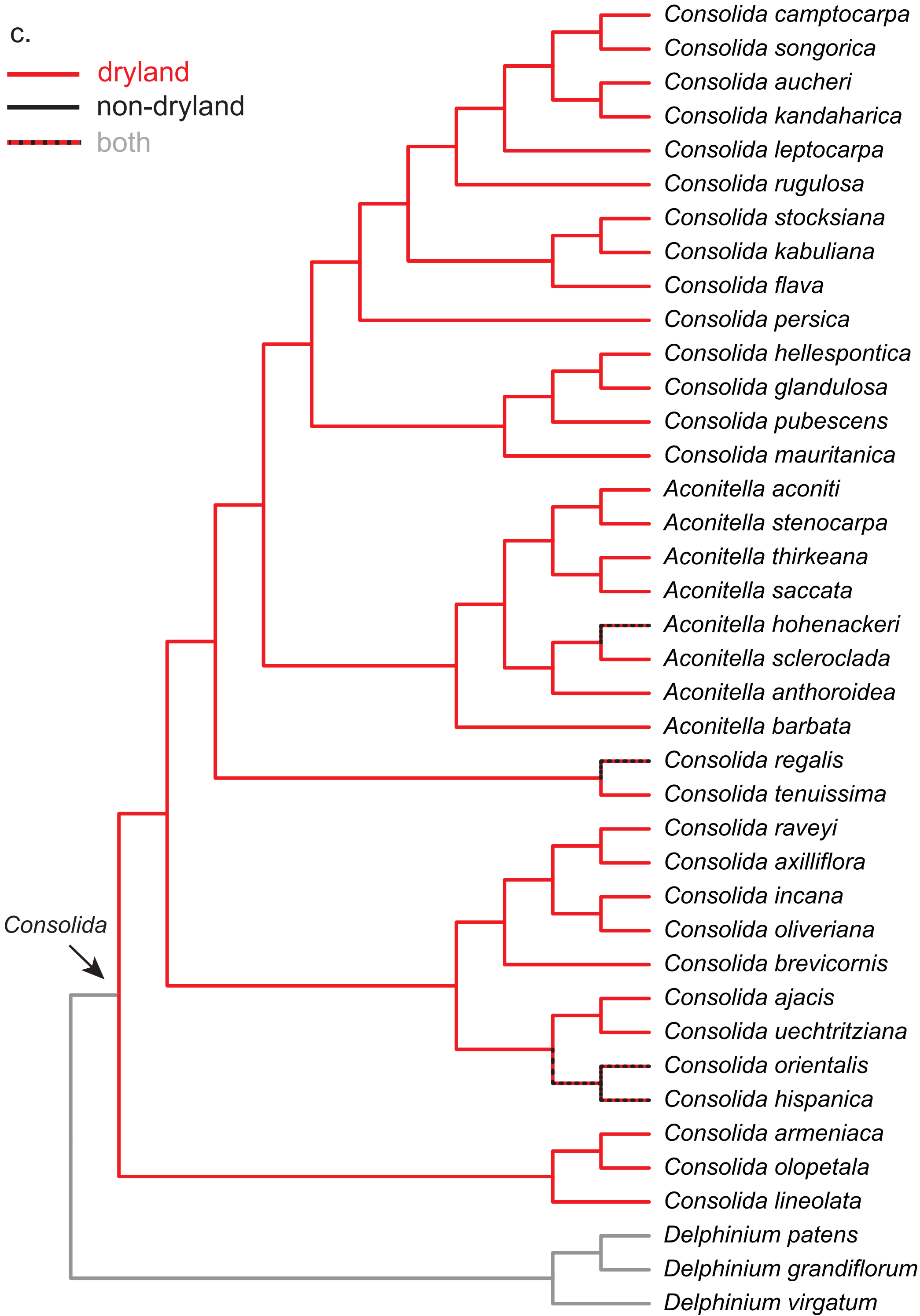
c.
dryland
non-dryland
both
Consolida camptocarpa
Consolida songorica
Consolida aucheri
Consolida kandaharica
Consolida leptocarpa
Consolida rugulosa
Consolida stocksiana
Consolida kabuliana
Consolida flava
Consolida persica
Consolida hellespontica
Consolida glandulosa
Consolida pubescens
Consolida mauritanica
Aconitella aconiti
Aconitella stenocarpa
Aconitella thirkeana
Aconitella saccata
Aconitella hohenackeri
Aconitella scleroclada
Aconitella anthoroidea
Aconitella barbata
Consolida regalis
Consolida tenuissima
Consolida raveyi
Consolida axilliflora
Consolida incana
Consolida oliveriana
Consolida brevicornis
Consolida ajacis
Consolida uechtritziana
Consolida orientalis
Consolida hispanica
Consolida armeniaca
Consolida olopetala
Consolida lineolata
Delphinium patens
Delphinium grandiflorum
Delphinium virgatum
Consolida

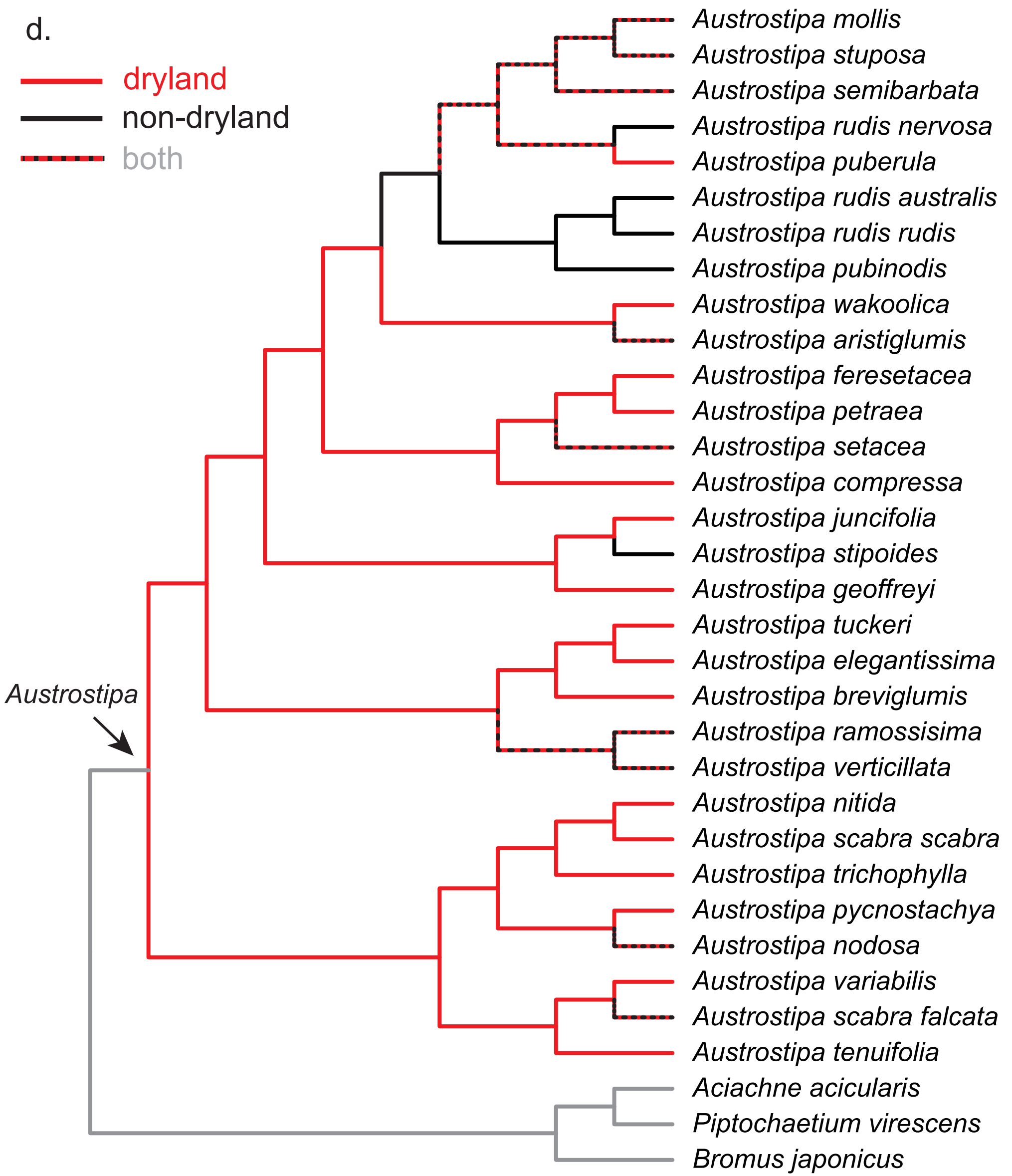
d.
dryland
non-dryland
both
Austrostipa
Austrostipa mollis
Austrostipa stuposa
Austrostipa semibarbata
Austrostipa rudis nervosa
Austrostipa puberula
Austrostipa rudis australis
Austrostipa rudis rudis
Austrostipa pubinodis
Austrostipa wakoolica
Austrostipa aristiglumis
Austrostipa feresetacea
Austrostipa petraea
Austrostipa setacea
Austrostipa compressa
Austrostipa juncifolia
Austrostipa stipoides
Austrostipa geoffreyi
Austrostipa tuckeri
Austrostipa elegantissima
Austrostipa breviglumis
Austrostipa ramossisima
Austrostipa verticillata
Austrostipa nitida
Austrostipa scabra scabra
Austrostipa trichophylla
Austrostipa pycnostachya
Austrostipa nodosa
Austrostipa variabilis
Austrostipa scabra falcata
Austrostipa tenuifolia
Aciachne acicularis
Piptochaetium virescens
Bromus japonicus

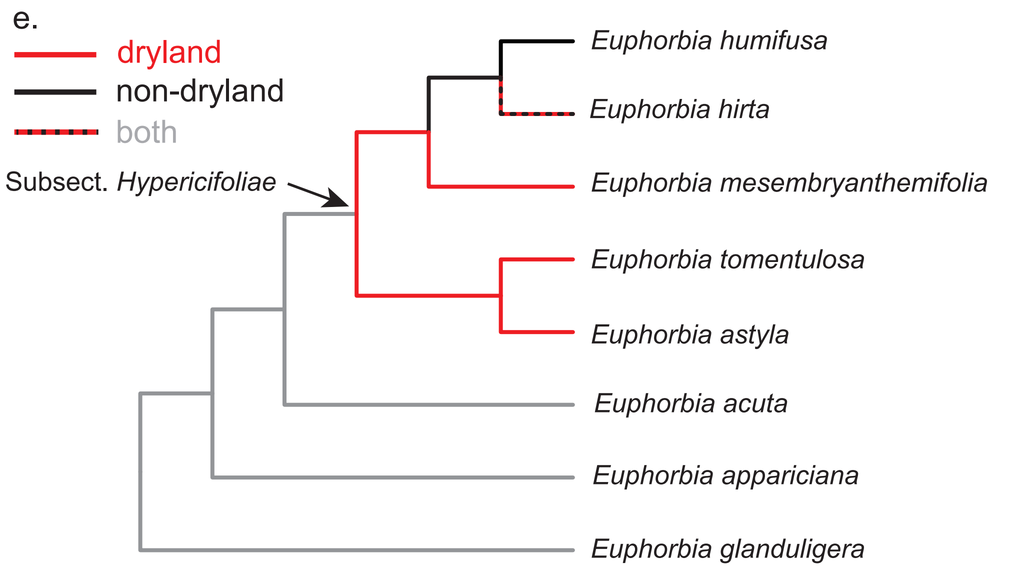
e.
dryland
non-dryland
both
Subsect. *Hypericifoliae*
Euphorbia humifusa
Euphorbia hirta
Euphorbia mesembryanthemifolia
Euphorbia tomentulosa
Euphorbia astyla
Euphorbia acuta
Euphorbia appariciana
Euphorbia glanduligera
